# Health-Related Quality of Life in Patients With Locally Advanced Gastric Cancer Undergoing Perioperative or Postoperative Adjuvant S-1 Plus Oxaliplatin With D2 Gastrectomy: A Propensity Score-Matched Cohort Study

**DOI:** 10.3389/fonc.2022.853337

**Published:** 2022-04-04

**Authors:** Jianhong Yu, Zaozao Wang, Zhexuan Li, Ying Liu, Yingcong Fan, Jiabo Di, Ming Cui, Jiadi Xing, Chenghai Zhang, Hong Yang, Zhendan Yao, Nan Zhang, Lei Chen, Maoxing Liu, Kai Xu, Fei Tan, Pin Gao, Xiangqian Su

**Affiliations:** ^1^ Department of Gastrointestinal Surgery IV, Key Laboratory of Carcinogenesis and Translational Research, Peking University Cancer Hospital and Institute, Beijing, China; ^2^ Key Laboratory of Carcinogenesis and Translational Research, Department of Cancer Epidemiology, Peking University Cancer Hospital and Institute, Beijing, China

**Keywords:** HRQOL, quality of life, gastric cancer, perioperative chemotherapy, SOX (S-1 + oxaliplatin)

## Abstract

**Background:**

Some high-quality clinical trials have proven the efficacy and safety of perioperative and postoperative S-1 with oxaliplatin (peri-SOX and post-SOX) for patients with locally advanced gastric cancer (LAGC) undergoing D2 gastrectomy. However, little is known about how health-related quality of life (HRQOL) changes over time in patients receiving peri-SOX or post-SOX chemotherapy.

**Methods:**

A prospective observational cohort (NCT04408859) identified 151 eligible patients with LAGC who underwent D2 gastrectomy with at least six cycles of peri-SOX or post-SOX chemotherapy from 2018 to 2020. HRQOL was assessed using the EROTC QLQ-C30 and its gastric module, QLQ-STO22, at indicated measurements, including the baseline, 1st, 3rd, 6th and 12th month after initiation of therapy. Baseline characteristics, therapeutic effects, and longitudinal HRQOL were compared between the peri-SOX and post-SOX groups after propensity score matching. HRQOL changes over time and the risk factors for scales with severe deterioration were further analyzed.

**Results:**

No statistically significant differences in longitudinal HRQOL were observed between patients in the peri-SOX and post-SOX groups, with comparable surgical outcomes and adverse chemotherapy events. Scales of social functioning, abnormal taste, and anxiety improved earlier in the peri-SOX group than in the post-SOX group. Score changes in both groups indicated that general deterioration and slower recovery usually occurred in the scales of physical, social, and role functioning, as well as symptoms of fatigue, reflux, diarrhea, and anxiety.

**Conclusion:**

Peri-SOX showed a longitudinal HRQOL comparable to post-SOX in patients with LAGC who underwent D2 gastrectomy. The peri-SOX group had better performance in social functioning, abnormal taste, and anxiety at some measurements.

## Introduction

Gastric cancer (GC) is the fourth and third leading cause of cancer-related deaths globally and in China, respectively (1, 2). The 5-year overall survival rate for patients with GC has improved after years of effort; however, it is still not more than 35% in China, which is partly attributable to nearly 80% of patients being diagnosed at advanced stages at initial hospitalization (3). Radical gastrectomy with adequate lymphadenectomy constitutes the mainstay of GC treatment, while recurrence and metastasis after complete resection illustrate that surgery alone cannot achieve a cure for gastric malignancy (4–6).

Adjuvant chemotherapy is now an essential treatment for Asian patients with locally advanced gastric cancer (LAGC) after surgery, since its encouraging therapeutic efficacy has been confirmed by the ACTS-GC and CLASSIC trials (7, 8). The MAGIC, FNCLCC, and FLOT4 trials conducted in Europe recommended perioperative chemotherapy, namely, neoadjuvant chemotherapy, followed by surgery and adjuvant chemotherapy as standard treatment for European patients with LAGC (9–11). Despite ethnic differences, the inconsistencies in chemotherapy regimens and administration durations between the East and West provide clues for healthcare providers to explore a more optimized chemotherapy strategy.

In East Asia, fluoropyrimidine-based chemotherapies are the preferred treatment for GC, with capecitabine plus oxaliplatin (CapOx) and S-1 with oxaliplatin (SOX) regimens as Grade I recommendations for postoperative adjuvant chemotherapy, and SOX for neoadjuvant chemotherapy in China (12–14). With an increasing number of perioperative chemotherapy-related studies in Asia, RESOLVE and PRODIGY have been devoted to comparing different sequences and combinations of perioperative chemotherapy with postoperative chemotherapy (15, 16). Results from the RESOLVE trial demonstrated a superior 3-year disease-free survival rate of perioperative SOX (59.4%) over adjuvant CapOx (51.1%) and a comparable efficacy between adjuvant SOX (56.5%) and adjuvant CapOx for T4 stage LAGC patients (15). Xue et al. also reported a comparable 5-year overall survival between perioperative SOX (peri-SOX) and postoperative SOX (post-SOX) after D2 resection (17). The gradually improved survival rate due to innovations in medical treatment has led to an increased number of long-term GC survivors, whose health-related quality of life (HRQOL) has attracted more attention.

As an important indicator of psychosocial burden emphasized by oncologists and psychologists, HRQOL has become an essential endpoint for evaluating the efficacy and impact of cancer treatment (18). Since gastrectomy may lead to nutritional and functional problems, while chemotherapy may result in nausea, vomiting, or fatigue, majority of studies related to HRQOL mainly focus on the effects of different surgical approaches or reconstruction methods in patients with resectable GC and the impact of palliative therapy on patients with unresectable or recurrent GC (19–32). The impact of the resection scope as well as the application of minimally invasive methods on patients’ HRQOL has greatly attracted the attention of surgeons. The recovery of patients receiving distal or proximal gastrectomy has been proven to be superior to total gastrectomy, as less functional and symptomatic problems will occur after subtotal resection (19–22). The potential HRQOL benefit of the laparoscopic approach compared to open surgery has also been reported for long-term follow-up (23, 24). Roux-en-Y reconstruction may lead to less severe gastrointestinal symptoms than Billroth I and II methods, although controversies still exist in this area (25–27). For patients with unresectable or recurrent GC, HRQOL is an important indicator for evaluating the therapeutic efficacy of different palliative chemotherapy regimens (28, 29). With the emergence of immunotherapy and targeted therapy, maintaining HRQOL for a longer time is important in palliative therapy using new drugs (30–32). Studies comparing the effects of different chemotherapy modalities on the HRQOL of patients with resectable upper alimentary tract carcinoma have mainly focused on esophageal or junctional cancers (33, 34). The ARTIST 2 trial recently reported a similar HRQOL in patients with LAGC undergoing D2 resection with adjuvant S-1, SOX, or SOX plus chemoradiotherapy (35). Studies evaluating HRQOL in patients with LAGC on pre- or perioperative chemotherapy are limited. Satake et al. assessed the neurotoxicity-related QOL in patients with LAGC receiving neoadjuvant SOX chemotherapy in a phase I study (36); however, additional data on HRQOL were not reported in its subsequent phase II trial (37). Studies comparing the impact of peri-SOX and post-SOX on the HRQOL of patients with LAGC undergoing D2 resection could hardly be found.

Therefore, the HRQOL changes in patients with LAGC receiving peri-SOX or post-SOX over time were evaluated in detail. Moreover, surgical outcomes, adverse chemotherapy events, changes in between-group and within-group longitudinal HRQOL in the peri-SOX and post-SOX groups, and risk factors for seriously deteriorated HRQOL scales with slow recovery were comprehensively analyzed in the present study.

## Methods

### Study Design and Patients

An observational cohort study focusing on HRQOL in patients with LAGC receiving perioperative versus postoperative chemotherapy with D2 gastrectomy (NCT04408859) was launched at the Department of Gastrointestinal Surgery IV of Peking University Cancer Hospital in 2018. From April 2018 to March 2020, 330 patients with LAGC undergoing D2 resection with SOX chemotherapy were administered self-reported questionnaires. This study was approved by the Research Ethics Committee of Peking University Cancer Hospital and Institute, and written informed consent was obtained from each participant.

The inclusion criteria were as follows: ① 18–80 years old; ② histologically confirmed gastric adenocarcinoma; ③ preoperatively diagnosed as clinical tumor stage II–III (T2-3N+M0 and T4NanyM0) according to the eighth edition of the American Joint Committee on Cancer (38); ④ patients receiving either peri-SOX or post-SOX chemotherapy, with two or three cycles of neoadjuvant SOX administered 3–6 weeks before surgery and the remaining cycles delivered 4–8 weeks after surgery for the peri-SOX group, or at least 6 cycles of adjuvant SOX started no later than 2 months after surgery for the post-SOX group. To identify the net effect of peri-SOX and post-SOX modalities on patients’ HRQOL, participants were excluded if ① the completed SOX chemotherapy was less than 6 cycles in total; ② questionnaires’ responses were less than twice in all measurements; ③ patients received postoperative chemoradiation with SOX; and ④ adjuvant SOX was commenced more than 3 months after surgery.

### HRQOL Measurements

Questionnaires collecting patient-reported outcomes were distributed and returned online by a dedicated research nurse through the “Wenjuanxing” platform, a professional and widely accepted platform in China for questionnaire surveys. HRQOL surveys were administered to patients during the first hospitalization before treatment and in 1st, 3rd, 6th, and 12th month after the initiation of therapy. Further follow-up phone calls were to be given if the completed questionnaire was not sent back within 2 weeks.

HRQOL of LAGC patients was assessed using the Chinese versions of the previously validated European Organization for Research and Treatment of Cancer Quality of Life Questionnaire-Core 30 (EORTC QLQ-C30) and EORTC QLQ-Gastric Cancer Module QLQ-STO22 (39–41). As a structured questionnaire for cancer self-management, the QLQ-C30 is generally suitable for all cancer patients, with widely accepted reliability and effectiveness (42). As a supplement to QLQ-C30, QLQ-STO22 further measures the HRQOL of GC patients at all pathological stages who underwent surgical resection, palliative intervention, endoscopic remission, or palliative chemotherapy (43, 44). Raw HRQOL questionnaire scores were linearly transformed from 0 to 100, with a higher score representing a better level of functional or global health status, but indicating a worse level of the symptom scales or items (45).

### Statistical Analysis

To minimize possible selection bias in this observational study, between-treatment differences were adjusted using the propensity score matching (PSM) approach, with age, sex, body mass index (BMI), ASA score, comorbidities, clinical T stage, and clinical N stage as covariates, in consideration of clinical relevance (46). LAGC patients receiving peri-SOX or post-SOX were matched through a 1:1 nearest-neighbor algorithm using a caliper width setting as 0.2 of a standard deviation of the logit of the propensity score (47). Absolute standardized differences (ASD) were calculated to assess covariate balance in the matched sample (48).

The comparison of baseline characteristics and clinical outcomes between the peri-SOX and post-SOX groups was performed using Student’s *t*-test or Mann–Whitney *U* test for continuous variables after assessing normality, and chi-squared analysis or Fisher’s exact test for categorical variables.

Baseline HRQOL scores between the two groups were compared using Student’s *t*-test. As a set of longitudinal data with repeated measures obtained from the same population, a linear mixed model was applied to explore any differential effect on HRQOL between peri-SOX and post-SOX groups over time and to examine differences between both groups at each follow-up measurement (the 1st, 3rd, 6th, and 12th month after the initiation of therapy) (49). If treatment effects over time were comparable between the peri-SOX and post-SOX groups, the HRQOL scores of both groups were combined to analyze the longitudinal effects on each scale of the questionnaire. Due to the allowance of the linear mixed model that analyzed a longitudinal dataset with missing-at-random data (50–53), all QOL scores of eligible patients were included in this analysis, regardless of any incomplete single item or drop-out during follow-up. After standardization of both predictor and outcome variables, the treatment effect was estimated using a linear mixed model that included the treatment arm, time, and treatment-by-time interaction as fixed effect factors and a random effect on patients, adjusted for baseline HRQOL with an autoregressive covariance structure of the first order. The beta estimates in the mixed modeling procedure demonstrated standardized differential effects between treatment groups over time or longitudinal effects, which were represented as Cohen’s *d* (CD) effect sizes for clinical relevance after standardized comparison (33). CD values of 0.2, 0.5, and 0.8 were cutoff points indicating small, medium, and considerable clinical significance, respectively (54). To correct for multiple testing, Bonferroni correction was applied according to the number of comparisons performed. Sensitivity analyses were performed to examine attrition bias by comparing the baseline characteristics and surgical outcomes between those who completed all study visits and those who did not. Moreover, the longitudinal effects on HRQOL between the two groups were examined using data before and after multiple imputation.

To present the alterations in HRQOL over time more clearly, scores in each domain were classified as “improved”, “stable”, or “deteriorated” with clinical relevance at each follow-up measurement, defined as at least 10-point score changes from baseline (55, 56). For the purpose of exploring the associations of baseline and treatment factors with several HRQOL domains, which represented severe deterioration and slow recovery at the 12th month after the beginning of therapy, Mantel-Haenszel chi-squared tests were applied to screen out single variables potentially influencing HRQOL changes in the univariate analysis. Data before PSM were used because the matched data might not represent its original source owing to the selectively reduced sample size. Factors with *p* < 0.20 in the univariate analysis were selected and further entered into the multivariate analysis by using ordinal logistic regression models after validating proportional odds assumptions by the test of parallel lines (57). The significance level was set at *p* < 0.05, except for multiple comparisons, and all analyses were conducted using the Statistical Package for the Social Sciences software (version 25.0; SPSS, Chicago, IL, USA).

## Results

### Characteristics of Study Population

**Figure 1:**
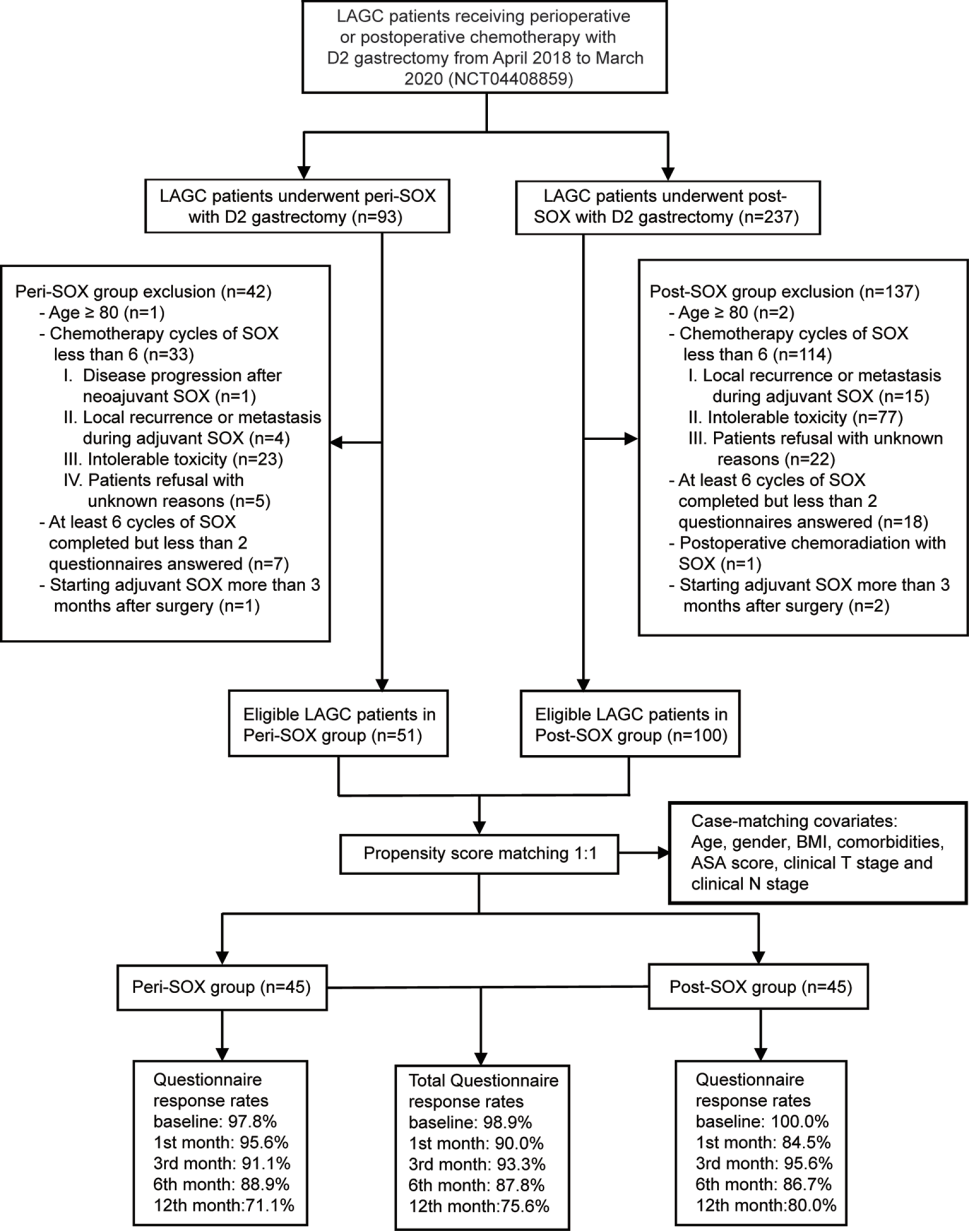
STROBE flow diagram illustrating the eligibility screening of LAGC patients receiving D2 gastrectomy with peri-SOX or post-SOX chemotherapy from April 2018 to March 2020, in a propensity score-matched observational cohort study.

**Figure 2:**
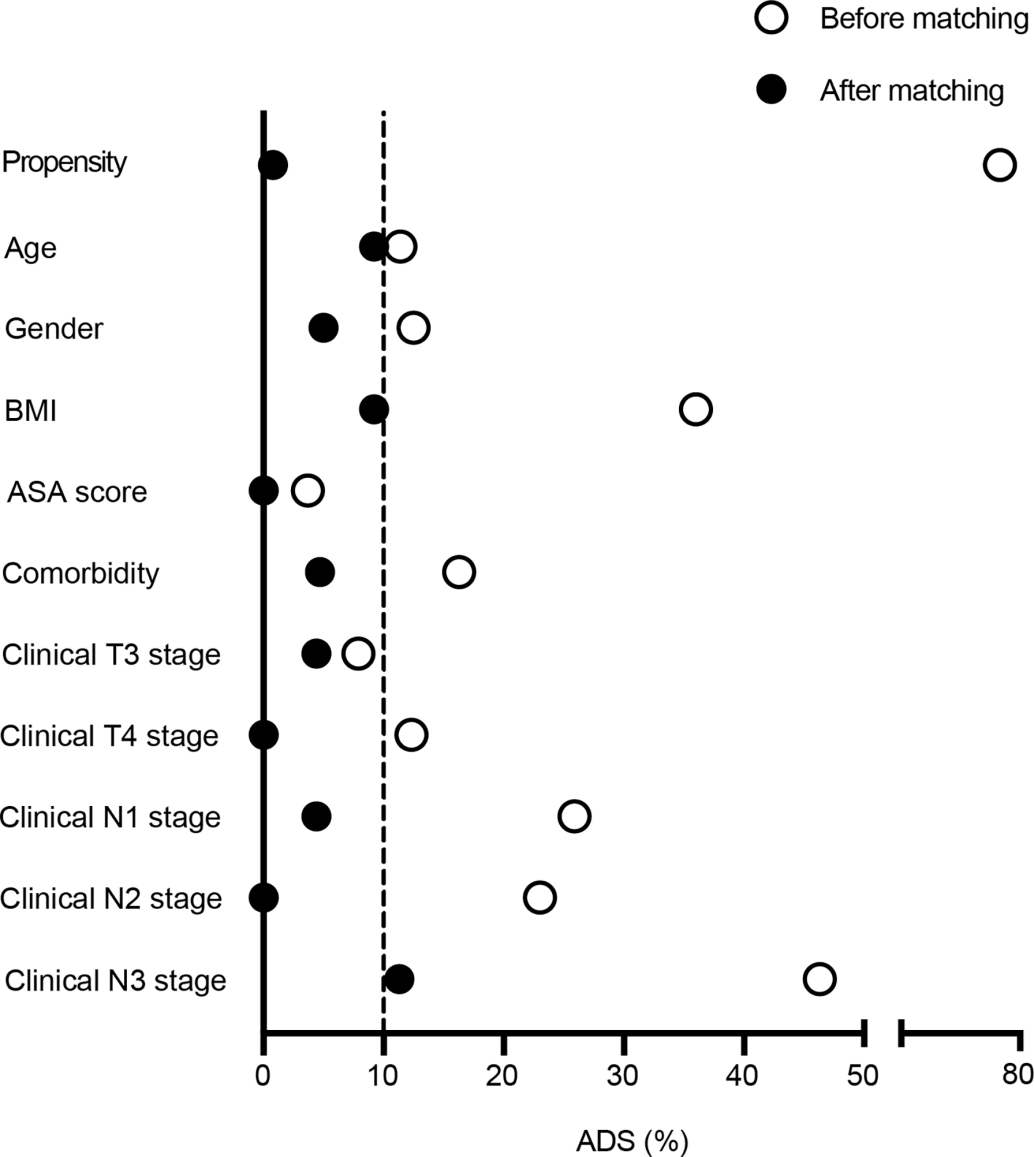
Absolute standardized differences were calculated for baseline variables before and after 1:1 propensity score matching. Labels in *y*-axis were the baseline characteristics of eligible LAGC patients, and the scatterplot represented absolute standardized differences of propensity scores before and after PSM.

This observational cohort included 151 eligible patients from April 2018 to March 2020, with 51 in the peri-SOX group and 100 in the post-SOX group, respectively. As shown in the STROBE flow diagram (**Figure 1**), 33 patients (35.5%) in the peri-SOX group and 114 patients (48.1%) in the post-SOX group could not complete at least six cycles of SOX chemotherapy (chi-squared test, *p =* 0.038). Seven patients in the peri-SOX and eighteen patients in the post-SOX group were unwilling to respond to the questionnaires at least twice, even if they had completed the indicated cycles of chemotherapy. The baseline characteristics of these two arms were comparable except for BMI (*p =* 0.032) and clinical nodal stage (*p =* 0.025), which were reported to affect the HRQOL of cancer patients, and acted as potential confounders in this study (58–61). After PSM, baseline characteristics were evenly distributed between the peri-SOX and post-SOX groups, with 45 patients in each group (**Table 1**). The ASD in the matched sample further proved a good balance after PSM (**Figure 2**).

**Table 1:** Baseline characteristics of eligible LAGC patients in peri-SOX and post-SOX groups before and after PSM.

	Original cohort		*P* ^B^	Matched cohort ^A^		*p* ^B^
	Peri-SOX (*n* = 51)	Post-SOX (*n* = 100)		Peri-SOX (*n* = 45)	Post-SOX (*n* = 45)	
Age			0.413			0.428
Median (IQR)	60.0 (54.0–64.0)	61.0 (55.3–66.0)		60.0 (54.0–64.0)	57.0 (53.0–64.0)	
BMI			**0.032**			0.843
Mean (SD)	22.9 ± 3.1	24.0 ± 2.9		23.5 ± 2.8	23.3 ± 2.4	
Gender			0.481			0.499
Male	38 (74.5%)	69 (69.0%)		32 (71.1%)	29 (64.4%)	
Female	13 (25.5%)	31 (31.0%)		13 (28.9%)	16 (35.6%)	
Comorbidity			0.357			0.499
None	35 (68.6%)	61 (61.0%)		29 (64.4%)	32 (71.1%)	
≥1 condition	16 (33.8%)	39 (39.0%)		16 (35.6%)	13 (28.9%)	
ASA score			0.834			0.334
I	7 (13.7%)	15 (15.0%)		7 (15.6%)	4 (8.9%)	
II	44 (86.3%)	85 (85.0%)		38 (84.4%)	41 (91.1%)	
Clinical tumor stage			0.204			0.410
T2	3 (5.9%)	16 (16.0%)		3 (6.7%)	5 (11.1%)	
T3	26 (51.0%)	47 (47.0%)		23 (51.1%)	17 (37.8%)	
T4	22 (43.1%)	37 (37.0%)		19 (42.2%)	23 (51.1%)	
Clinical nodal stage			**0.025**			0.908
N0	6 (11.8%)	27 (27.0%)		6 (13.3%)	7 (15.6%)	
N1	24 (47.1%)	34 (34.0%)		20 (44.4%)	17 (37.8%)	
N2	19 (37.3%)	26 (26.0%)		17 (37.8%)	18 (40.0%)	
N3	2 (3.9%)	13 (13.0%)		2 (4.4%)	3 (6.7%)	

Peri-SOX, perioperative chemotherapy with S-1 and oxaliplatin; Post-SOX, postoperative chemotherapy with S-1 and oxaliplatin; IQR, interquartile range; SD, standard deviation; BMI, body mass index; ASA, American Society of Anesthesiologists.

Data are presented as n (%) unless otherwise stated.

^A^The covariates used for propensity score matching include age, BMI, gender, comorbidity, ASA score, clinical tumor stage, and clinical nodal stage.

^B^Bold p-values indicate statistical significance (p < 0.05).

### Surgical Outcomes and Adverse Events of Chemotherapy

As reported in **Table 2**, intraoperative outcomes and postoperative complication rates were comparable between the peri-SOX and post-SOX arms after PSM. No severe complications or mortality was reported within 30 days of surgery. Adverse events of chemotherapy were similar between the two groups, suggesting a comparable impact of the perioperative or postoperative chemotherapy modality. Nearly 90% of patients with LAGC who completed at least six cycles of SOX had side effects, although adverse events of grades 4–5 were not observed in either group. The number of patients with grade 1–2 adverse events was similar between the two arms. Grade 3 adverse events occurred in 35.6% of patients in the peri-SOX group and 37.8% of patients in the post-SOX group, with neutropenia being the most common symptom, followed by leukopenia (**Table S1**).

**Table 2:** Intraoperative outcomes, postoperative complications, and adverse events of chemotherapy between peri-SOX and post-SOX groups after PSM.

	Peri-SOX (*n* = 45)	Post-SOX (*n* = 45)	*p*
**Intraoperative outcomes**			
Gastrectomy			0.371
Distal	17 (37.8%)	13 (28.9%)	
Total	28 (62.2%)	32 (71.1%)	
Type of surgery			0.140
Open	27 (60.0%)	20 (44.4%)	
Laparoscopy-assisted	18 (40.0%)	25 (55.6%)	
Operative time (min)			0.465
Median (IQR)	223.0 (180.0–275.0)	235.0 (207.5–272.5)	
Intraoperative blood loss (ml)			0.341
Median (IQR)	65.0 (50.0–135.0)	90.0 (50.0–140.0)	
No. of harvested lymph node			0.622
Median (IQR)	33.0 (24.5–38.0)	34.0 (25.5–39.5)	
Radical resection	45 (100.0%)	43 (95.6%)	0.494
Mortality in 30 days	0	0	
**Postoperative complications**			
Total complications	12 (26.7%)	15 (33.3%)	0.490
Abdomen infection	2 (4.4%)	1 (2.2%)	
Anastomotic leak	1 (2.2%)	1 (2.2%)	
Pancreatic fistula	0	1 (2.2%)	
Duodenal stump leak	0	1 (2.2%)	
Bleeding in abdomen	0	1 (2.2%)	
Ileus	1 (2.2%)	1 (2.1%)	
Delayed gastric emptying	0	1 (2.2%)	
Ascites	3 (6.7%)	2 (4.4%)	
Pulmonary infection	2 (4.4%)	3 (6.7%)	
Pleural effusion	2 (4.4%)	2 (4.4%)	
Wound infection	1 (2.2%)	1 (2.2%)	
Clavien–Dindo classification			0.918
None	33 (73.3%)	30 (66.7%)	
I	3 (6.7%)	4 (8.9%)	
II	6 (13.3%)	7 (15.5%)	
III	3 (6.7%)	4 (8.9%)	
IV and V	0	0	
**Adverse events of SOX chemotherapy**			0.819
None	5 (11.1%)	4 (8.9%)	
Grade 1	11 (24.4%)	8 (17.8%)	
Grade 2	13 (28.9%)	16 (35.6%)	
Grade 3	16 (35.6%)	17 (37.8%)	
Grades 4 and 5	0	0	
Dose modification of SOX regimen			
S-1	6 (13.3%)	7 (15.6%)	0.764
Oxaliplatin	11 (24.4%)	12 (26.7%)	0.809

Peri-SOX, perioperative chemotherapy with S-1 and oxaliplatin; Post-SOX, postoperative chemotherapy with S-1 and oxaliplatin; IQR, interquartile range.

Data are presented as n (%) unless otherwise stated.

### Changes of HRQOL in Peri-SOX and Post-SOX Groups

The overall questionnaire response rates throughout this 1-year follow-up period decreased slightly from 98.9% at baseline, to 75.6% in the 12th month after the initiation of therapy, even under rigorous follow-up by the research nurse. Similar response rates were observed in both populations who had completed sufficient chemotherapy cycles with good compliance (**Figure 1**).

**Figure 3:**
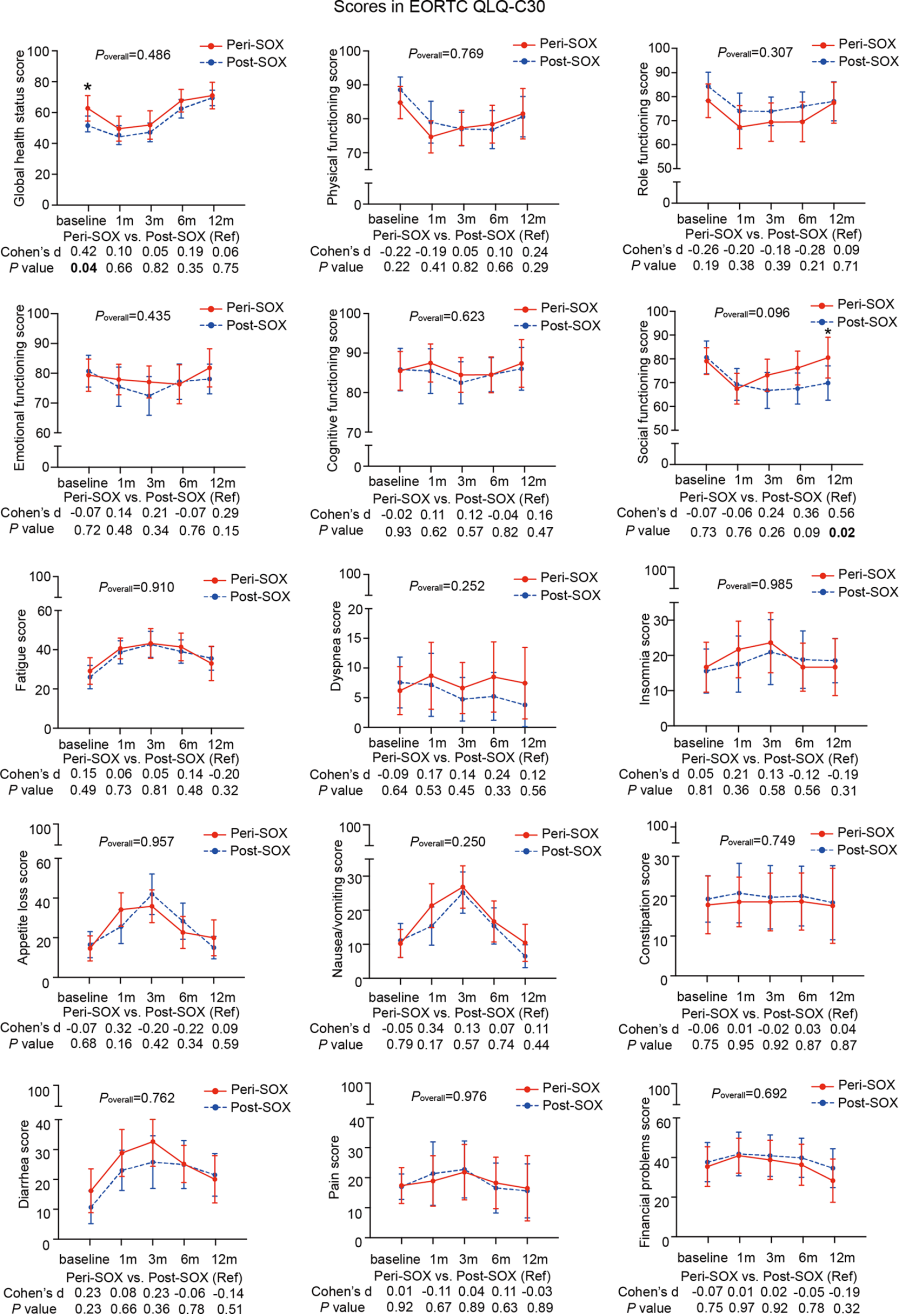
Mean scores with 95% confidence interval (CI) over time of each scale or item in the HRQOL questionnaire, EORTC QLQ-C30, according to the treatment group. *P*_overall_ represented statistical values of the longitudinal comparison between the peri-SOX and post-SOX groups. Between-group differences of HRQOL scores at baseline and at each follow-up measurement were also analyzed by the linear mixed model; Cohen’s *d* (CD) effect size and *p*-values were listed correspondingly under the line graph. Bold font and * indicated statistically significant difference with *p* < 0.05.

**Figure 4:**
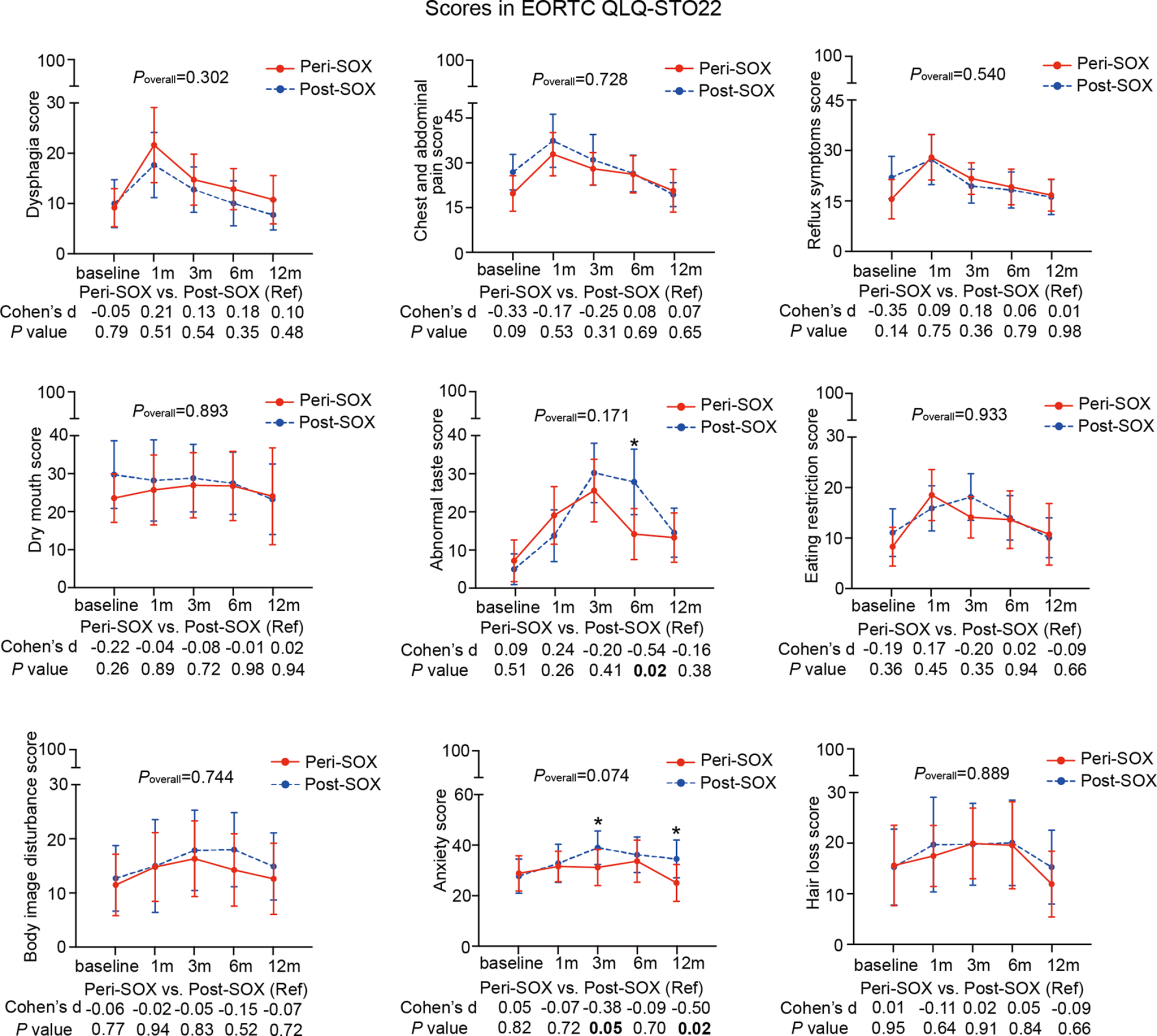
Mean scores with 95% confidence interval (CI) over time of each scale or item in the HRQOL questionnaire, EORTC QLQ-STO22, according to the treatment group. *P*_overall_ represented statistical values of the longitudinal comparison between the peri-SOX and post-SOX groups. Between-group differences of HRQOL scores at baseline and at each follow-up time point were also analyzed by the linear mixed model; Cohen’s *d* (CD) effect size and *p*-values were listed correspondingly under the line graph. Bold font and * indicated statistically significant difference with *p* < 0.05.

The changing trajectories of mean HRQOL scores in each scale or item of the EORTC QLQ-C30 and STO22 at the indicated measurement points throughout the longitudinal study are presented in **Figures 3**, **4** for the peri-SOX and post-SOX groups. No statistically significant differences were observed in any scale of the questionnaires between the two groups over time. Similar baseline features or surgical outcomes were observed between those who completed all study visits and those who did not, indicating that the lost data were missing at random (**Tables S2, S3**). Results from linear mixed modeling before and after multiple imputations showed comparable overall trends in HRQOL trajectories between the groups (**Table S4**), suggesting that no attrition bias were observed in this study.

The baseline HRQOL scores were similar in all scales and items between the peri-SOX and post-SOX groups, except for global health status, which was significantly better in the peri-SOX group, with more than a ten-point difference. The changing trajectories were similar with the passage of time between the peri-SOX and post-SOX groups in the majority of HRQOL scales, with some exceptions (**Figures 3**, **4**). Social functioning deteriorated and reached the lowest point 1 month after baseline and gradually recovered afterwards in both groups, while improvement in social functioning in the peri-SOX group was more obvious than in the post-SOX group, with a remarkable difference in the 12th month (CD, 0.56; *p =* 0.02). The symptom scores for abnormal taste were much lower in the peri-SOX arm than in the post-SOX arm in the 6th month, indicating a milder tasting problem in the peri-SOX group (CD, −0.54; *p =* 0.02). The anxiety scores in the peri-SOX group remained stable during treatment and follow-up; meanwhile, in the post-SOX group, this symptom deteriorated continuously and reached the worst level by the 3rd month, with a slow recovery thereafter. Patients in the peri-SOX group were remarkably less anxious than those in the post-SOX group by the 3rd (CD, −0.38; *p =* 0.05) and 12th month (CD, −0.50; *p =* 0.02) after the commencement of therapy.

### Overall Trends of HRQOL Along With Time

The longitudinal changes in HRQOL scores in each scale or item between baseline and follow-up measurements were calculated with data from both groups using linear mixed models, as changes in HRQOL over time could not be affected by the sequence of chemotherapy. As shown in **Table 3**, the overall trends in 10 out of 24 domains remained stable over time, compared to their baseline levels, including cognitive functioning; symptoms of dyspnea, insomnia, constipation, pain, and financial problems in the QLQ-C30 questionnaire; and dry mouth, body image, anxiety, and hair loss in the QLQ-STO22 questionnaire.

**Table 3:** Longitudinal effects of both peri-SOX and post-SOX treatments on scales or items in EORTC QLQ-C30 and QLQ-STO22 questionnaires of LAGC patients with D2 gastrectomy over time.

	Follow-up after baseline (months)																			
	1			3					6				12							
	CD ^A^	95% CI	*p* ^B^	CD ^A^		95% CI	*p* ^B^		CD ^A^	95% CI	*p* ^B^		CD ^A^				95% CI			*p* ^B^
QLQ-C30																				
Global health status	−0.45	(−0.70, −0.20)	**<0.001**	−0.37	(−0.63, −0.11)		**0.005**	0.30		(0.03, 0.56)		0.028		0.59	(0.31, 0.87)			**<0.001**		
Functioning scales																				
Physical functioning	−0.62	(−0.83, −0.40)	**<0.001**	−0.59	(−0.83, −0.35)		**<0.001**		−0.57	(−0.82, −0.31)		**<0.001**	−0.28			(−0.55, −0.01)			0.045	
Role functioning	−0.45	(−0.67, −0.24)	**<0.001**	−0.43	(−0.68, −0.18)		**0.001**		−0.39	(−0.66, −0.13)		**0.003**	−0.14			(−0.42, 0.14)			0.318	
Emotional functioning	−0.20	(−0.40, −0.00)	0.045	−0.31	(−0.54, −0.07)		**0.010**		−0.21	(−0.46, 0.04)		0.103	0.01			(−0.26, 0.28)			0.956	
Cognitive functioning	0.06	(−0.17, 0.30)	0.609	−0.14	(−0.39, 0.11)		0.283		−0.13	(−0.39, 0.13)		0.329	0.06			(−0.21, 0.33)			0.672	
Social functioning	−0.55	(−0.76, −0.34)	**<0.001**	−0.47	(−0.71, −0.23)		**<0.001**		−0.38	(−0.63, −0.12)		**0.004**	−0.19			(−0.46, 0.08)			0.172	
Symptom scales																				
Fatigue	0.58	(0.38, 0.78)	**<0.001**	0.75	(0.52, 0.98)		**<0.001**		0.62	(0.38, 0.86)		**<0.001**	0.29			(0.04, 0.54)			0.024	
Dyspnea	0.06	(−0.19, 0.31)	0.647	−0.07	(−0.35, 0.20)		0.604		−0.01	(−0.29, 0.28)		0.985	−0.13			(−0.43, 0.17)			0.383	
Insomnia	0.14	(−0.10, 0.37)	0.255	0.26	(0.01, 0.52)		0.048		0.07	(−0.20, 0.33)		0.631	−0.01			(−0.29, 0.27)			0.946	
Appetite loss	0.55	(0.31, 0.79)	**<0.001**	0.89	(0.62, 1.15)		**<0.001**		0.36	(0.08, 0.64)		**0.011**	0.04			(−0.26, 0.33)			0.815	
Nausea/vomiting	0.44	(0.19, 0.68)	**<0.001**	0.86	(0.60, 1.13)		**<0.001**		0.28	(0.01, 0.56)		0.042	−0.18			(−0.47, 0.11)			0.214	
Constipation	−0.04	(−0.27, 0.19)	0.729	−0.08	(−0.34, 0.18)		0.557		−0.06	(−0.33, 0.20)		0.642	−0.14			(−0.42, 0.14)			0.317	
Diarrhea	0.49	(0.27, 0.71)	**<0.001**	0.67	(0.42, 0.91)		**<0.001**		0.50	(0.24, 0.76)		**<0.001**	0.30			(0.03, 0.57)			0.028	
Pain	0.10	(−0.16, 0.37)	0.444	0.22	(−0.07, 0.50)		0.133		0.03	(−0.26, 0.32)		0.821	−0.08			(−0.38, 0.22)			0.597	
Financial problems	0.15	(−0.04, 0.33)	0.126	0.13	(−0.09, 0.34)		0.247		0.06	(−0.18, 0.29)		0.638	−0.18			(−0.42, 0.06)			0.144	
QLQ-STO22																				
Dysphagia	0.67	(0.42, 0.93)	**<0.001**	0.25	(−0.02, 0.53)		0.071		0.11	(−0.17, 0.39)		0.442	−0.09			(−0.39, 0.21)			0.554	
Chest and abdominal pain	0.56	(0.31, 0.81)	**<0.001**	0.30	(0.02, 0.57)		0.033		0.16	(−0.12, 0.44)		0.266	−0.21			(−0.51, 0.08)			0.158	
Reflux symptoms	0.51	(0.28, 0.74)	**<0.001**	0.10	(−0.16, 0.36)		0.463		−0.01	(−0.28, 0.27)		0.988	−0.19			(−0.48, 0.11)			0.215	
Dry mouth	0.02	(−0.24, 0.27)	0.887	0.03	(−0.25, 0.31)		0.818		0.01	(−0.29, 0.29)		0.991	−0.14			(−0.44, 0.16)			0.367	
Abnormal taste	0.45	(0.20, 0.70)	**<0.001**	0.94	(0.67, 1.21)		**<0.001**		0.66	(0.38, 0.93)		**<0.001**	0.35			(0.08, 0.63)			0.013	
Eating restriction	0.49	(0.25, 0.73)	**<0.001**	0.44	(0.17, 0.70)		**0.001**		0.31	(0.03, 0.58)		0.030	−0.02			(−0.32, 0.27)			0.872	
Body image	0.17	(−0.08, 0.41)	0.184	0.24	(−0.04, 0.51)		0.088		0.19	(−0.09, 0.47)		0.190	0.08			(−0.22, 0.38)			0.595	
Anxiety	0.19	(−0.01, 0.39)	0.064	0.29	(0.06, 0.53)		0.015		0.29	(0.42, 0.54)		0.022	0.02			(−0.25, 0.29)			0.886	
Hair loss	0.17	(−0.07, 0.41)	0.170	0.20	(−0.06, 0.47)		0.131		0.17	(−0.10, 0.45)		0.221	−0.05			(−0.34, 0.24)			0.745	

CD, Cohen’s d; Peri-SOX, perioperative chemotherapy with S-1 and oxaliplatin; Post-SOX, postoperative chemotherapy with S-1 and oxaliplatin; QLQ-C30, Quality of Life Questionnaire-Core 30; QLQ-STO22, Quality of Life Questionnaire-Gastric Cancer Module 22; CI, confidence interval; LAGC, locally advanced gastric cancer.

^A^Cohen’s d effect size is derived from the beta estimate in the mixed linear modeling procedure after standardization of both outcome and predictor variables, with baseline score of scales or items as reference for each comparison.

^B^Statistical significance is set at p < 0.0125 after a Bonferroni correction due to the main analyses including four comparisons for each scale or item; bold p-values indicate statistical significance.

The global health status, physical functioning, role functioning, and social functioning in the QLQ-C30 questionnaire, and symptoms of dysphagia, chest and abdominal pain, reflux, and eating restriction in the QLQ-STO22 questionnaire deteriorated to the worst level in the first month after baseline, and their recovery trajectories were different. Symptoms of dysphagia, chest and abdominal pain, and reflux improved by the 3rd month, and remained stable thereafter. Global health status and eating restriction returned to a comparable level as the baseline till the 6th month, and the global health status became even significantly better than the pre-treatment level by the 12th month. Physical, role, and social functioning recovered slowly, and the significant differences compared to baseline values disappeared by the 12th month. Fatigue, appetite loss, nausea, and vomiting, in addition to diarrhea in the QLQ-C30 questionnaire and abnormal taste in the QLQ-STO22 questionnaire deteriorated significantly 1 month after the initiation of therapy and reached the worst level by the 3rd month. Symptoms of nausea and vomiting were relieved by the 6th month, while fatigue, appetite loss, diarrhea, and abnormal taste were still problematic and only returned to baseline levels by the 12th month (**Table 3**).

**Figure 5:**
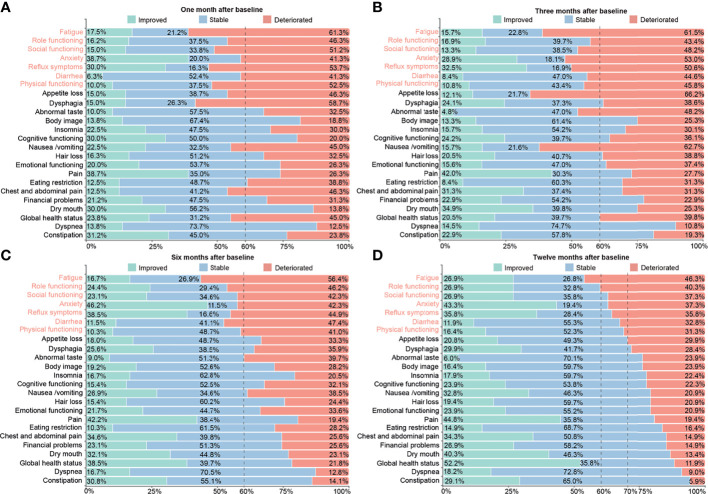
Proportions of patients with clinically significant statuses classified as “improved”, “stable”, and “deteriorated” in the scales or items of the QLQ-C30 and QLQ-STO22 questionnaires at each follow-up measurement, including **(A)** 1 month, **(B)** 3 months, **(C)** 6 months, and **(D)** 12 months after baseline. Status with clinical significance was defined as at least 10-point changes relative to the baseline. Titles of scales or items were colored in red if more than 40% of patients showed deteriorated status by the 1st, 3rd, and 6th month after initiation of therapy, and more than 30% of patients still had worrisome symptoms by the 12th month.

To clearly describe the dynamic changes of patients’ conditions over time from the clinical point of view, each HRQOL scale was classified into “improved”, “stable”, or “deteriorated” with corresponding ratios according to the clinically meaningful difference of HRQOL (55, 56). More than 40% of patients presented “deteriorated” in scales of physical, social, and role functioning, as well as symptoms of fatigue, reflux, diarrhea, and anxiety in the 1st, 3rd, and 6th month after baseline, and at least 30% patients remained troublesome even by the 12th month among these scales, with fatigue ranking with the highest deterioration rate at each follow-up measurement (**Figure 5**).

Further analyses were conducted to explore risk factors associated with the seven scales exhibiting general deterioration and slower recovery in this longitudinal study. Twelve clinically relevant factors were initially involved in finding single variables related to HRQOL change in the concerned scales, and independent risk factors were further confirmed using subsequent ordinal logistic regression (**Table 4** and **Tables S5–S10**). The results indicated that after completion of gastrectomy and chemotherapy, baseline characteristics and therapeutic factors affected the HRQOL of patients. As presented in **Table 4**, a higher BMI can induce more severe fatigue. As we set the scores of fatigue in patients with BMI < 25 as a reference, the odds ratio (OR) for relief of fatigue in patients with BMI ≥ 25 was 0.81-fold than that in the reference group. Similar results were observed in the other scales. Higher BMI and comorbidity ≥1 were negatively correlated with the alleviation of anxiety (**Table S5**). Recovery of physical functioning was poorer in patients with adverse events of chemotherapy (**Table S6**). In addition, the scope of gastrectomy also correlated with recovery from diarrhea, with better improvement in patients undergoing distal gastrectomy than in those receiving total gastrectomy (**Table S7**). The involved 12 variables were not associated with score changes in role functioning, social functioning, and reflux symptoms (**Tables S8–10**), indicating their weakened roles in these scales 1 year later.

**Table 4:** Univariate and multivariate analyses of associations between clinically relevant factors and changes of fatigue of LAGC patients before PSM in the 12th month after the initiation of therapy.

Variables	Changes of fatigue (%)			Total	Univariate analysis *p* ^A^	Multivariate analysis		
	Deteriorated	Stable	Improved			OR	95% CI	*p* ^B^
Age					0.062			
<60	25 (47.1%)	19 (35.9%)	9 (17.0%)	53		Ref		
≥60	30 (52.6%)	10 (17.5%)	17 (29.9%)	57		1.82	(0.98, 1.42)	0.079
Gender					0.355			
Male	40 (52.6%)	21 (27.6%)	15 (19.8%)	76				
Female	15 (44.1%)	8 (23.5%)	11 (32.4%)	34				
BMI					0.100			
<25	31 (43.7%)	19 (26.8%)	21 (29.5%)	71		Ref		
≥25	24 (61.5%)	10 (25.6%)	5 (12.9%)	39		0.81	(0.68, 0.97)	**0.019**
ASA score					0.712			
I	10 (58.8%)	4 (23.5%)	3 (17.7%)	17				
II	45 (48.4%)	25 (26.9%)	23 (24.7%)	93				
Comorbidities					0.979			
None	37 (49.3%)	20 (26.7%)	18 (24.0%)	75				
≥1	18 (51.4%)	9 (25.7%)	8 (22.9%)	35				
Clinical T stage					0.463			
T2	7 (43.8%)	5 (31.2%)	4 (25.0%)	16				
T3	29 (58.0%)	12 (24.0%)	9 (18.0%)	50				
T4	19 (43.1%)	12 (27.3%)	13 (29.6%)	44				
Clinical N stage					0.896			
N0	15 (53.6%)	7 (25.0%)	6 (21.4%)	28				
N1	21 (48.8%)	10 (23.3%)	12 (27.9%)	43				
N2	16 (51.6%)	8 (25.8%)	7 (22.6%)	31				
N3	3 (37.5%)	4 (50.0%)	1 (12.5%)	8				
Sequence of chemotherapy					0.684			
Post-SOX	39 (52.7%)	19 (25.7%)	16 (21.6%)	74				
Peri-SOX	16 (44.4%)	10 (27.8%)	10 (27.8%)	36				
Gastrectomy					0.416			
Total	33 (52.4%)	18 (28.6%)	12 (19.0%)	63				
Distal	22 (46.8%)	11 (23.4%)	14 (29.8%)	47				
Surgical method					0.549			
Open	20 (48.8%)	13 (31.7%)	8 (19.5%)	41				
Laparoscopy	35 (50.7%)	16 (23.2%)	18 (26.1%)	69				
Surgical complications					0.508			
No	49 (51.6%)	24 (25.3%)	22 (23.1%)	95				
Yes	6 (40.0%)	5 (33.3%)	4 (26.7%)	15				
Adverse events of chemotherapy					0.257			
No	2 (33.3%)	1 (16.7%)	3 (50.0%)	6				
Yes	53 (51.0%)	28 (26.9%)	23 (22.1%)	104				

Data presented as n (%).

BMI, body mass index; ASA, American Society of Anesthesiologists; OR, odds ratio; CI, confidence interval; Ref, reference group.

^A^Bold p-values indicate statistical significance (p < 0.05) according to the Mantel-Haenszel Chi-squared tests.

^B^Bold p-values indicate statistical significance (p < 0.05) according to the ordinal logistic regression analysis.

## Discussion

With good application prospects of the SOX regimen in either neoadjuvant or adjuvant chemotherapy, several high-quality clinical trials are being conducted to evaluate the surgical safety, efficacy, and survival benefit of D2 resection plus SOX with different sequences or cycles in patients with LAGC (15, 37, 62, 63). In addition to surgical and oncological endpoints, the HRQOL is another instrument used to evaluate the effect of cancer treatment on patients’ lives. In this longitudinal study, peri-SOX treatment had a comparable impact on HRQOL over time as the post-SOX group in LAGC patients with sufficient chemotherapy cycles. At some follow-up points, the scores of HRQOL in social functioning, abnormal taste, and anxiety were much better in the peri-SOX group than in the post-SOX group, indicating that the peri-SOX modality has a positive influence on few HRQOL scales.

Since a small sample size would lead to reduced statistical power, CD values with relatively small or moderate clinical effect sizes might not have statistical significance in this observational study (64). To describe the HRQOL changes in detail, clinical effect size rather than simply *p*-value was considered. Despite comparable comorbidity and ASA scores at baseline between these two groups, significantly superior general health statuses were reported by patients in the peri-SOX group than in the post-SOX group. Milder symptoms of chest and abdominal pain, reflux, and dry mouth were also observed in the peri-SOX arm, with only slightly worse physical and role functioning before treatment. This phenomenon is in accordance with real-world clinical practice. With proven therapeutic effects of neoadjuvant and adjuvant SOX chemotherapy with D2 resection, both are standard treatments for stage II–III LAGC (including both EGJ and non-EGJ cancers), except for a slight difference in the recommended level (13). Neoadjuvant chemotherapy could effectively degrade the tumor stage but destroy the anatomical dissection plane due to tissue fibrotic changes, which might lead to increased surgical difficulties. Therefore, the sequence of chemotherapy administration is a comprehensive decision considering both tumor degradation demand and surgical risks. In patients with similar general conditions, doctors usually tend to recommend that individuals with better self-feelings and milder gastric symptoms receive neoadjuvant chemotherapy before surgery.

In the first month after the initiation of therapy, symptoms of insomnia, appetite loss, nausea and vomiting, dysphagia, and abnormal taste, as well as role functioning deteriorated slightly in the peri-SOX group compared to the post-SOX group, which may be due to the administration of neoadjuvant chemotherapy in the peri-SOX group at this time point. Although with some skipping of CD values, scales of emotional functioning, social functioning, symptoms of appetite loss, abnormal taste, and anxiety improved considerably in the peri-SOX group than in the post-SOX group, with small clinical significance for at least two measurements. By the 12th month, scales of social functioning, physical functioning, emotional functioning, and symptoms of anxiety and fatigue showed small or moderate improvements in the peri-SOX group compared to the control group, which indicated a better recovery of patients in the peri-SOX group (**Figures 3**, **4**).

The global health status in both the peri-SOX and post-SOX groups improved gradually, from significantly worse than the baseline level to remarkably better than the baseline level, indicating the curative effectiveness of gastrectomy together with chemotherapy in patients with LAGC (**Table 3**). Most scales of HRQOL presented a significant deterioration in the first and third month after baseline, as treatments were given in this period for both groups. Hereafter, the deteriorated domains of HRQOL were generally relieved. Up until the 12th month, no HRQOL score was significantly lower than the baseline level (**Table 3**). However, some scales still showed more than 30% deterioration at this time point, which continued to worsen from the beginning of therapy and returned to the baseline very slowly (**Figure 5**). Independent risk factors for HRQOL domains with general deterioration and slower recovery were further analyzed. We found that some baseline and clinically relevant characteristics could contribute to the deterioration of the HRQOL scales. Elevated BMI at baseline was a high-risk factor that led to worsened HRQOL in anxiety and fatigue, as previously reported (58–60). Total gastrectomy as a clinically relevant factor was associated with unrelieved diarrhea, compared to distal gastrectomy, while adverse events of chemotherapy might prolong the recovery time of patients (**Table 4** and **Tables S5–S10**). As some symptoms are unavoidable due to therapeutic strategies, healthcare providers should pay much attention during treatment. Relevant problems should be fully informed to patients with LAGC before treatment. Furthermore, to minimize discomfort during this period, simultaneous supportive care should be provided if necessary.

This study had several limitations. First, we only included patients with LAGC who received at least six cycles of SOX in total. In a pilot study, we found that questionnaire response rates decreased significantly in patients unable to complete chemotherapy and led to a significant increase in missing data, which might influence the stability of the linear mixed model (53). In addition, the survival benefit was comparable between patients receiving no fewer than six cycles of SOX, but not in patients with fewer cycles (65, 66). Moreover, chemotherapy cycles affect the HRQOL of cancer patients (67, 68). Therefore, participants receiving insufficient cycles of SOX were excluded to explore the net effect of chemotherapy sequence on the HRQOL of patients with LAGC. However, approximately 30%–50% of patients with LAGC in China cannot complete sufficient cycles of chemotherapy (15, 63). Hence, the estimation of HRQOL in patients with less than six cycles of SOX should be done with caution because of the limited external validity of this study. Second, the long-term HRQOL of the peri-SOX and post-SOX groups could not be observed due to the 1-year follow-up. Third, although the reliability of this observational cohort study was increased by applying PSM, an underpowered risk of statistical analysis may have occurred because of the reduced sample size.

## Conclusion

In conclusion, peri-SOX or post-SOX chemotherapy modalities have little effect on the HRQOL of LAGC patients who receive D2 gastrectomy over time. The scales of social functioning, abnormal taste, and anxiety improve earlier in the peri-SOX group than in the post-SOX group at some follow-up measurements. General deterioration and slower recovery usually occur in scales of physical functioning, social functioning, role functioning, and symptoms of fatigue, reflux, diarrhea, and anxiety; thus, patients with LAGC with higher risk factors should be fully informed before commencement of either peri-SOX or post-SOX treatment.

## Data Availability Statement

The raw data supporting the conclusions of this article will be made available by the authors, without undue reservation.

## Ethics Statement

The studies involving human participants were reviewed and approved by the Research Ethics Committee of Peking University Cancer Hospital & Institute. The patients/participants provided their written informed consent to participate in this study.

## Author Contributions

JY and ZW drafted the protocol, extracted the data, performed the statistical analyses, and drafted the article. ZL performed the statistical analyses and directed statistical analyses. YL delivered and collected the questionnaire. YF, JD, MC, JX, CZ, HY, ZY, NZ, LC, ML, KX, FT, and PG drafted the protocol, participated in patient enrollment, and revised the manuscript. XS and ZW directed the writing, review, and revision of the article. JY, ZW, ZL, and YL have contributed equally to this work and share first authorship. All authors contributed to the article and approved the submitted version.

## Funding

This study was supported by the Capital’s Funds for Health Improvement and Research (CFH 2018-2-2153), the National Natural Science Foundation of China (nos. 82073357, 82171720, 81872022, and 81672439), and the Beijing Natural Science Foundation (no. 7162039).

## Conflict of Interest

The authors declare that the research was conducted in the absence of any commercial or financial relationships that could be construed as a potential conflict of interest.

## Publisher’s Note

All claims expressed in this article are solely those of the authors and do not necessarily represent those of their affiliated organizations, or those of the publisher, the editors and the reviewers. Any product that may be evaluated in this article, or claim that may be made by its manufacturer, is not guaranteed or endorsed by the publisher.
