# Supplementary material for: Health-Related Quality of Life in Patients With Locally Advanced Gastric Cancer Undergoing Perioperative or Postoperative Adjuvant S-1 Plus Oxaliplatin With D2 Gastrectomy: A Propensity Score-Matched Cohort Study
Source: Front Oncol. 2022 Apr 4;12:853337. doi: 10.3389/fonc.2022.853337 (PMC9013949; doi:10.3389/fonc.2022.853337)
Supplement: Supplementary file 1 [file DataSheet_1.docx]

**
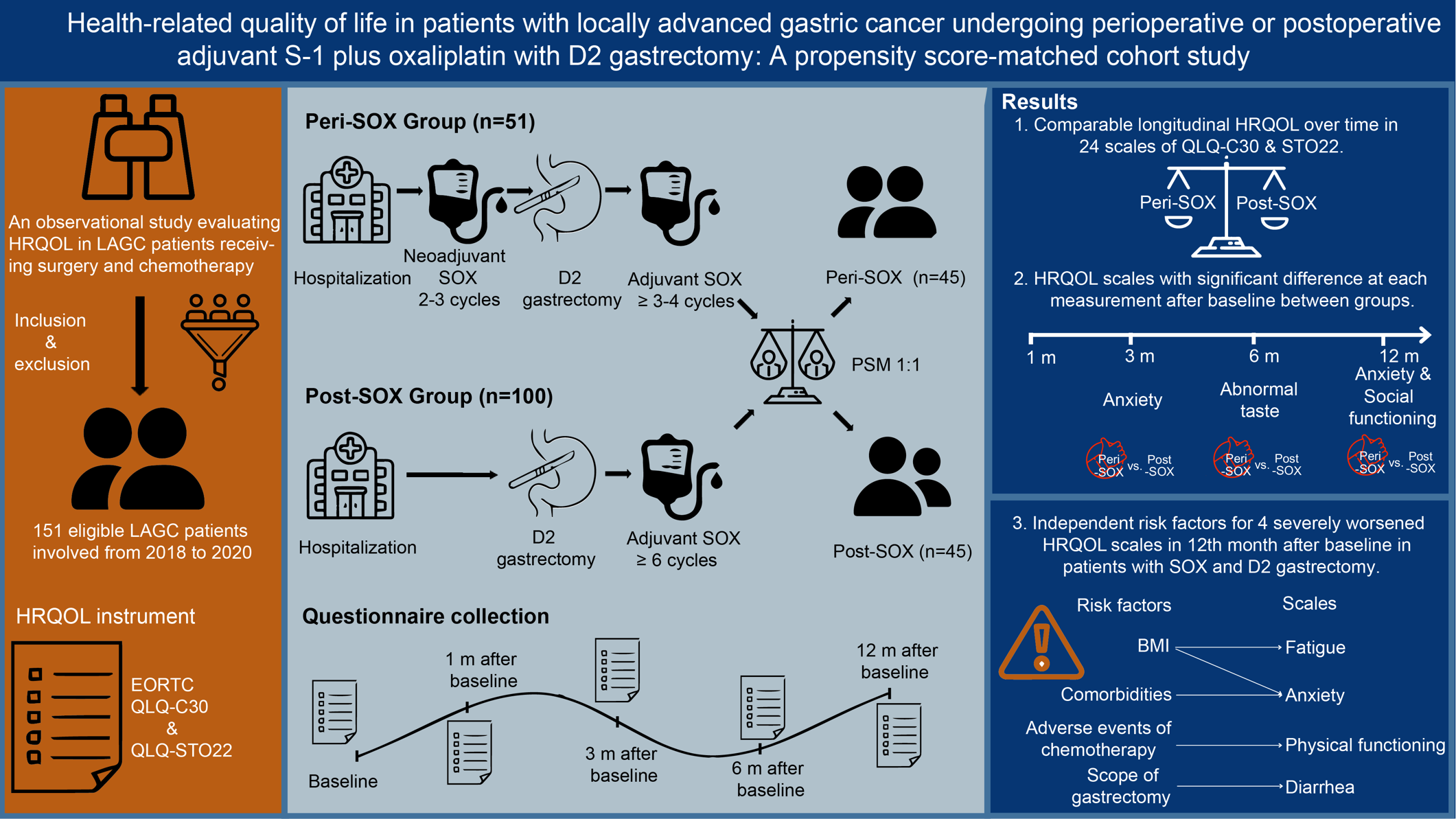
**

**Supplementary Figure 1.** Visual abstract to state the key question being addressed and provide a visual display of outcomes.

**Table S1**. Summary of adverse events.

|  | Peri-SOX group (n=45) | | |  | Post-SOX group (n=45) | | | *P* ^A^ |
| --- | --- | --- | --- | --- | --- | --- | --- | --- |
|  | Grade 1 | Grade 2 | Grade 3 |  | Grade 1 | Grade 2 | Grade 3 |  |
| All adverse events | 11 (24.4%) | 13 (28.9%) | 16 (35.6%) |  | 8 (17.8%) | 16 (35.6%) | 17 (37.8%) | 0.819 |
| Neutropenia | 6 (13.3%) | 14 (31.3%) | 12 (26.7%) |  | 5 (11.1%) | 16 (35.6%) | 10 (22.2%) | 0.931 |
| Leukopenia | 9 (20.0%) | 12 (26.7%) | 6 (13.3%) |  | 14 (31.3%) | 11 (24.4%) | 6 (13.3%) | 0.653 |
| Thrombocytopenia | 9 (20.0%) | 4 (8.9%) | 4 (8.9%) |  | 8 (17.8%) | 1 (2.2%) | 2 (4.4%) | 0.376 |
| Anaemia | 7 (15.6%) | 3 (6.7%) | 4 (8.9%) |  | 4 (8.9%) | 2 (4.4%) | 2 (4.4%) | 0.529 |
| ALT or AST increased | 6 (13.3%) | 1 (2.2%) | 0 |  | 7 (15.6) | 1 (2.2%) | 0 | 0.956 |
| Gastrointestinal symptoms | 12 (26.7%) | 8 (17.8%) | 1 (2.2%) |  | 8 (17.8%) | 9 (20.0%) | 5 (11.1%) | 0.315 |
| Neurotoxicity | 4 (8.9%) | 4 (8.9%) | 0 |  | 9 (20.0%) | 1 (2.2%) | 0 | 0.151 |

Abbreviations: Peri-SOX: perioperative chemotherapy with S-1 and oxaliplatin, Post-SOX: postoperative chemotherapy with S-1 and oxaliplatin, ALT: alanine aminotransferase, AST: aspartate aminotransferase.

Data are presented as n (%).

Adverse events of grade 4 and grade 5 were not observed in this study.

^A^ Bold P values indicate statistical significance (P <0.05).

**Table S2**. Baseline characteristics of eligible LAGC patients with or without all study visits.

|  | Patients with all visits (n=57) | | Patients without all visits (n=33) | *P* ^A^ |
| --- | --- | --- | --- | --- |
| Age | | | | 0.159 |
| Median (IQR) | 57.0 (50.5-64.0) | 59.0 (55.5-64.0) | |  |
| BMI | | | | 0.539 |
| Mean (SD) | 23.3±2.6 | 23.6±2.6 | |  |
| Gender | | | | 0.445 |
| Male | 37 (64.9%) | 24 (72.7%) | |  |
| Female | 20 (35.1%) | 9 (27.3%) | |  |
| Comorbidity | | | | 0.522 |
| None | 40 (70.2%) | 21 (63.6%) | |  |
| ≥1condition | 17 (29.8%) | 12 (36.4%) | |  |
| ASA score | | | | 0.490 |
| I | 8 (14.0%) | 3 (9.1%) | |  |
| II | 49 (86.0%) | 30 (90.9%) | |  |
| Clinical tumor stage | | | | 0.324 |
| T2 | 6 (10.5%) | 2 (6.1%) | |  |
| T3 | 22 (38.6%) | 18 (54.6%) | |  |
| T4 | 29 (50.9%) | 13 (39.4%) | |  |
| Clinical nodal stage | | | | 0.994 |
| N0 | 8 (14.0%) | 5 (15.2%) | |  |
| N1 | 24 (42.1%) | 13 (39.4%) | |  |
| N2 | 22 (38.6%) | 13 (39.4%) | |  |
| N3 | 3 (5.3%) | 2 (6.1%) | |  |

Abbreviations: IQR: interquartile range, SD: standard deviation, ASA: American Society of Anesthesiologists; BMI: body mass index.

Data are presented as n (%) unless otherwise stated.

^A^ Bold *P* values indicate statistical significance (*P* <0.05).

**Table S3**. Intraoperative outcomes, postoperative complications and adverse events of chemotherapy for eligible LAGC patients with or without all study visits.

|  | Patients with all visits (n=57) | Patients without all visits (n=33) | *P* ^A^ |
| --- | --- | --- | --- |
| **Intraoperative outcomes** | | | |
| Gastrectomy | | | 0.643 |
| Distal | 20 (35.1%) | 10 (30.3%) |  |
| Total | 37 (64.9%) | 23 (69.7%) |  |
| Type of surgery | | | 0.919 |
| Open | 30 (52.6%) | 17 (51.5%) |  |
| Laparoscopy-assisted | 27 (47.4%) | 16 (48.5%) |  |
| Operative time (min) | | | 0.580 |
| Median (IQR) | 230.0 (192.5-272.5) | 230.0 (180.0-272.5) |  |
| Intraoperative blood loss (ml) | | | 0.058 |
| Median (IQR) | 65.0 (50.0-95.0) | 90.0 (52.5-150.0) |  |
| No. of harvested lymph node | | | 0.100 |
| Median (IQR) | 34.0 (26.0-41.5) | 30.0 (23.0-37.0) |  |
| Radical resection | 56 (98.3%) | 32 (96.7%) | 0.692 |
| Mortality in 30 days | 0 | 0 |  |
| **Postoperative complications** | | | |
| Total complications | 19 (33.3%) | 8 (24.2%) | 0.364 |
| Abdomen infection | 2 (3.5%) | 1 (3.0%) | 0.903 |
| Anastomotic leak | 1 (1.8%) | 1 (3.0%) | 0.692 |
| Pancreatic fistula | 1 (1.8%) | 0 | 0.444 |
| Duodenal stump leak | 1 (1.8%) | 0 | 0.444 |
| Bleeding in abdomen | 1 (1.8%) | 0 | 0.444 |
| Ileus | 1 (1.8%) | 1 (3.0%) | 0.692 |
| Delayed gastric emptying | 0 | 1 (3.0%) | 0.186 |
| Ascites | 4 (7.0%) | 1 (3.0%) | 0.426 |
| Pulmonary infection | 3 (5.3%) | 2 (6.1%) | 0.874 |
| Pleural effusion | 3 (5.3%) | 1 (3.0%) | 0.620 |
| Wound infection | 2 (3.5%) | 0 | 0.277 |
| Clavien-Dindo classification | | | 0.841 |
| None | 38 (66.7%) | 25 (75.8%) |  |
| I | 5 (8.8%) | 2 (6.1%) |  |
| II | 9 (15.8%) | 4 (12.1%) |  |
| III | 5 (8.8%) | 2 (6.1%) |  |
| IV & V | 0 | 0 |  |
| **Adverse events of SOX chemotherapy** | | | 0.219 |
| None | 3 (5.3%) | 6 (18.2%) |  |
| Grade 1 | 14 (24.6%) | 5 (15.2%) |  |
| Grade 2 | 19 (33.3%) | 10 (30.3%) |  |
| Grade 3 | 21 (36.8%) | 12 (36.4%) |  |
| Grade 4 & 5 | 0 | 0 |  |

Abbreviations: IQR: interquartile range.

Data are presented as n (%) unless otherwise stated.

^A^ Bold *P* values indicating statistical significance (*P* <0.05).

**Table S4**. Statistical values before and after multiple imputation comparing longitudinal treatment effect between peri-SOX and post-SOX groups on scales or items in the EORTC QLQ-C30 and QLQ-STO22 questionnaires of LAGC patients with D2 gastrectomy.

|  | | before imputation | | After multiple imputations | | | | | | | | | | | | | |
| --- | --- | --- | --- | --- | --- | --- | --- | --- | --- | --- | --- | --- | --- | --- | --- | --- | --- |
|  |  |  |  | 1^st^ Imputation | | 2^nd^ imputation | | | 3^rd^ imputation | | 4^th^ imputation | | 5^th^ imputation | | Pooled imputation by a meta-analysis | | |
|  | Estimate Coefficient ^A^, 95%CI | | *P* ^B^ | Estimate Coefficient ^A^, 95%CI | *P* ^B^ | Estimate Coefficient ^A^, 95%CI | | *P* ^B^ | Estimate Coefficient ^A^, 95%CI | *P* ^B^ | Estimate Coefficient ^A^, 95%CI | *P* ^B^ | Estimate Coefficient ^A^, 95%CI | *P* ^B^ | Estimate Coefficient ^A^, 95%CI | *P* ^B^ | |
| QLQ-C30 | | | | | | | | | | | | | | | | | |
| Global health status | | 0.07  (-0.12,0.26) | 0.486 | 0.02  (-0.17,0.21) | 0.841 | -0.01  (-0.20,0.19) | 0.966 | | 0.01  (-0.19,0.20) | 0.979 | 0.04  (-0.16,0.24) | 0.690 | 0.04  (-0.16,0.23) | 0.699 | 0.02  (-0.18,0.22) | | 0.850 |
| Functioning scales | | | | | | | | | | | | | | | | | |
| Physical functioning | | 0.03  (-0.19,0.25) | 0.769 | -0.02  (-0.23,0.19) | 0.857 | 0.01  (-0.18,0.21) | 0.908 | | 0.02  (-0.18,0.23) | 0.819 | 0.02  (-0.19,0.23) | 0.842 | 0.02  (-0.18,0.22) | 0.861 | 0.01  (-0.19,0.22) | | 0.916 |
| Role functioning | | -0.12  (-0.35,0.11) | 0.307 | -0.13  (-0.35,0.09) | 0.244 | -0.12  (-0.33,0.09) | 0.262 | | -0.11  (-0.32,0.11) | 0.326 | -0.10  (-0.33,0.12) | 0.371 | -0.10  (-0.32,0.12) | 0.362 | -0.11  (-0.33,0.11) | | 0.313 |
| Emotional functioning | | 0.09  (-0.14,0.33) | 0.435 | 0.04  (-0.18,0.26) | 0.714 | 0.07  (-0.15,0.28) | 0.547 | | 0.09  (-0.14,0.31) | 0.449 | 0.13  (-0.09,0.36) | 0.245 | 0.14  (-0.08,0.36) | 0.218 | 0.09  (-0.15,0.33) | | 0.445 |
| Cognitive functioning | | 0.05  (-0.15,0.24) | 0.623 | 0.01  (-0.17,0.19) | 0.917 | 0.06  (-0.13,0.24) | 0.546 | | 0.05  (-0.13,0.24) | 0.573 | 0.10  (-0.08,0.29) | 0.267 | 0.10  (-0.09,0.28) | 0.310 | 0.06  (-0.13,0.27) | | 0.533 |
| Social functioning | | 0.19  (-0.03,0.42) | 0.096 | 0.16  (-0.05,0.38) | 0.131 | 0.14  (-0.07,0.35) | 0.193 | | 0.15  (-0.06,0.37) | 0.166 | 0.17  (-0.04,0.38) | 0.112 | 0.16  (-0.05,0.37) | 0.141 | 0.16  (-0.06,0.37) | | 0.147 |
| Symptom scales | | | | | | | | | | | | | | | | | |
| Fatigue | | 0.01  (-0.19,0.21) | 0.910 | 0.05  (-0.14,0.23) | 0.628 | 0.02  (-0.16,0.21) | 0.818 | | 0.03  (-0.16,0.21) | 0.771 | 0.01  (-0.19,0.20) | 0.987 | 0.02  (-0.17,0.21) | 0.814 | 0.02  (-0.17,0.21) | | 0.806 |
| Dyspnea | | 0.13  (-0.09,0.35) | 0.252 | 0.15  (-0.06,0.35) | 0.156 | 0.12  (-0.09,0.33) | 0.271 | | 0.18  (-0.02,0.38) | 0.081 | 0.18  (-0.03,0.39) | 0.091 | 0.13  (-0.08,0.33) | 0.235 | 0.15  (-0.06,0.36) | | 0.169 |
| Insomnia | | 0.01  (-0.20,0.21) | 0.985 | -0.01  (-0.21,0.19) | 0.940 | -0.03  (-0.23,0.17) | 0.780 | | 0.02  (-0.17,0.21) | 0.803 | 0.02  (-0.18,0.22) | 0.824 | 0.01  (-0.19,0.20) | 0.938 | 0.00  (-0.19,0.20) | | 0.972 |
| Appetite loss | | 0.01  (-0.21,0.22) | 0.957 | 0.07  (-0.13,0.28) | 0.494 | 0.04  (-0.15,0.24) | 0.638 | | 0.02  (-0.18,0.24) | 0.783 | 0.07  (-0.14,0.28) | 0.501 | 0.09  (-0.11,0.29) | 0.390 | 0.06  (-0.15,0.27) | | 0.565 |
| Nausea /vomiting | | 0.12  (-0.09,0.33) | 0.250 | 0.15  (-0.05,0.36) | 0.140 | 0.15  (-0.05,0.34) | 0.147 | | 0.14  (-0.06,0.34) | 0.165 | 0.16  (-0.04,0.35) | 0.123 | 0.14  (-0.07,0.34) | 0.185 | 0.15  (-0.05,0.35) | | 0.151 |
| Constipation | | 0.03  (-0.18,0.24) | 0.749 | 0.08  (-0.12,0.28) | 0.435 | 0.07  (-0.13,0.28) | 0.486 | | 0.05  (-0.16,0.26) | 0.629 | 0.07  (-0.13,0.28) | 0.471 | 0.07  (-0.13,0.28) | 0.476 | 0.07  (-0.13,0.28) | | 0.500 |
| Diarrhea | | 0.03  (-0.18,0.24) | 0.762 | 0.08  (-0.12,0.29) | 0.407 | 0.03  (-0.17,0.22) | 0.789 | | 0.02  (-0.18,0.22) | 0.814 | 0.01  (-0.18,0.20) | 0.935 | 0.06  (-0.13,0.26) | 0.523 | 0.04  (-0.17,0.25) | | 0.696 |
| Pain | | 0.01  (-0.21,0.20) | 0.976 | 0.02  (-0.18,0.22) | 0.853 | 0.06  (-0.14,0.25) | 0.568 | | -0.02  (-0.21,0.17) | 0.847 | -0.04  (-0.24,0.16) | 0.713 | -0.03  (-0.22,0.17) | 0.771 | -0.00  (-0.22,0.21) | | 0.986 |
| Financial problems | | -0.04  (-0.24,0.16) | 0.692 | 0.01  (-0.18,0.19) | 0.972 | -0.01  (-0.21,0.20) | 0.962 | | 0.02  (-0.18,0.22) | 0.847 | -0.03  (-0.22,0.17) | 0.788 | -0.08  (-0.27,0.11) | 0.419 | -0.02  (-0.23,0.19) | | 0.871 |
| QLQ-STO22 | | | | | | | | | | | | | | | | | |
| Dysphagia | | 0.11  (-0.10,0.33) | 0.302 | 0.14  (-0.09,0.36) | 0.228 | 0.20  (-0.01,0.41) | 0.057 | | 0.15  (-0.06,0.36) | 0.156 | 0.16  (-0.05,0.37) | 0.132 | 0.09  (-0.11,0.30) | 0.374 | 0.15  (-0.08,0.37) | | 0.198 |
| Chest and abdominal pain | | -0.04  (-0.26,0.18) | 0.728 | 0.09  (-0.12,0.30) | 0.395 | 0.04  (-0.16,0.25) | 0.671 | | 0.08  (-0.12,0.29) | 0.429 | 0.05  (-0.16,0.26) | 0.640 | -0.01  (-0.21,0.19) | 0.916 | 0.05  (-0.17,0.28) | | 0.652 |
| Reflux symptoms | | 0.08  (-0.17,0.32) | 0.540 | 0.05  (-0.19,0.30) | 0.660 | 0.08  (-0.15,0.31) | 0.486 | | 0.12  (-0.11,0.35) | 0.302 | 0.06  (-0.17,0.29) | 0.624 | 0.05  (-0.18,0.28) | 0.674 | 0.07  (-0.17,0.31) | | 0.553 |
| Dry mouth | | -0.02  (-0.25,0.21) | 0.893 | 0.04  (-0.18,0.25) | 0.718 | -0.03  (-0.25,0.18) | 0.747 | | 0.07  (-0.15,0.28) | 0.543 | 0.06  (-0.17,0.29) | 0.603 | -0.02  (-0.24,0.19) | 0.825 | 0.02  (-0.22,0.26) | | 0.860 |
| Abnormal taste | | -0.14  (-0.34,0.06) | 0.171 | -0.14  (-0.35,0.06) | 0.176 | -0.13  (-0.34,0.07) | 0.198 | | -0.13  (-0.33,0.07) | 0.218 | -0.09  (-0.30,0.12) | 0.394 | -0.12  (-0.33,0.09) | 0.260 | -0.12  (-0.33,0.09) | | 0.251 |
| Eating restriction | | -0.01  (-0.23,0.21) | 0.933 | 0.08  (-0.13,0.29) | 0.458 | 0.05  (-0.17,0.28) | 0.624 | | 0.06  (-0.17,0.28) | 0.623 | 0.07  (-0.14,0.28) | 0.506 | 0.02  (-0.18,0.23) | 0.812 | 0.06  (-0.16,0.27) | | 0.606 |
| Body image | | -0.04  (-0.26,0.19) | 0.744 | 0.01  (-0.19,0.21) | 0.930 | -0.07  (-0.28,0.15) | 0.533 | | -0.08  (-0.31,0.16) | 0.518 | -0.08  (-0.29,0.14) | 0.489 | -0.07  (-0.29,0.15) | 0.517 | -0.06  (-0.29,0.17) | | 0.629 |
| Anxiety | | -0.20  (-0.42,0.02) | 0.074 | -0.16  (-0.37,0.05) | 0.142 | -0.14  (-0.35,0.06) | 0.173 | | -0.16  (-0.37,0.05) | 0.123 | -0.19  (-0.40,0.01) | 0.064 | -0.16  (-0.36,0.04) | 0.114 | -0.16  (-0.37,0.04) | | 0.124 |
| Hair loss | | -0.02  (-0.24,0.20) | 0.889 | -0.04  (-0.24,0.17) | 0.726 | -0.03  (-0.23,0.16) | 0.735 | | -0.03  (-0.24,0.18) | 0.800 | -0.07  (-0.29,0.14) | 0.495 | -0.02  (-0.23,0.18) | 0.830 | -0.04  (-0.25,0.17) | | 0.717 |

Abbreviations: EORTC QLQ-C30: European Organization for Research and Treatment of Cancer Quality of Life Questionnaire-Core 30, QLQ-STO22: Quality of Life Questionnaire-Gastric Cancer Module 22, CI: confidence interval.

^A^ Estimate coefficient comparing treatment effect between peri-SOX and post-SOX (reference) was derived from the fixed effect of treatment arm in the mixed linear modeling procedure after standardization of both outcome and predictor variables.

^B^ Bold font *P* value indicating *P*<0.05.

**Table S5**. Univariate and multivariate analyses of associations between clinically relevant factors and changes of anxiety of LAGC patients before PSM in the 12th month after the initiation of therapy.

| Variables | Changes of anxiety (%) | | | Total | Univariate analysis  *P* ^A^ | Multivariate analysis | | |
| --- | --- | --- | --- | --- | --- | --- | --- | --- |
|  | Deteriorated | Stable | Improved |  |  | OR | 95% CI | *P* ^B^ |
| Age |  |  |  |  | **0.030** |  |  |  |
| < 60 | 18 (34.0%) | 13 (24.5%) | 22 (41.5%) | 53 |  | Ref |  |  |
| ≥ 60 | 28 (49.1%) | 4 (7.0%) | 25 (43.9%) | 57 |  | 0.92 | (0.43,1.95) | 0.819 |
| Gender |  |  |  |  | 0.058 |  |  |  |
| Male | 36 (47.4%) | 8 (10.5%) | 32 (42.1%) | 76 |  | Ref |  |  |
| Female | 10 (29.4%) | 9 (26.5%) | 15 (44.1%) | 34 |  | 1.04 | (0.45,2.39) | 0.924 |
| BMI |  |  |  |  | **0.026** |  |  |  |
| < 25 | 23 (32.4%) | 13 (18.3%) | 35 (49.3%) | 71 |  | Ref |  |  |
| ≥ 25 | 23 (59.0%) | 4 (10.2%) | 12 (30.8%) | 39 |  | 0.40 | (0.18,0.87) | **0.021** |
| ASA score |  |  |  |  | 0.852 |  |  |  |
| I | 8 (47.1%) | 2 (11.7%) | 7 (41.2%) | 17 |  |  |  |  |
| II | 38 (40.9%) | 15 (16.1%) | 40 (43.0%) | 93 |  |  |  |  |
| Comorbidities |  |  |  |  | **0.018** |  |  |  |
| None | 25 (33.3%) | 15 (20.0%) | 35 (46.7%) | 75 |  | Ref |  |  |
| ≥ 1 | 21 (60.0%) | 2 (5.7%) | 12 (34.3%) | 35 |  | 0.44 | (0.20,0.98) | **0.044** |
| Clinical T stage |  |  |  |  | 0.791 |  |  |  |
| T2 | 5 (31.3%) | 2 (12.5%) | 9 (56.3%) | 16 |  |  |  |  |
| T3 | 23 (46.0%) | 8 (16.0%) | 19 (38.0%) | 50 |  |  |  |  |
| T4 | 18 (40.9%) | 7 (15.9%) | 19(43.2%) | 44 |  |  |  |  |
| Clinical N stage |  |  |  |  | 0.924 |  |  |  |
| N0 | 11 (39.3%) | 3 (10.7%) | 14 (50.0%) | 28 |  |  |  |  |
| N1 | 19 (44.2%) | 9 (20.9%) | 15 (34.9%) | 43 |  |  |  |  |
| N2 | 13 (41.9%) | 5 (16.1%) | 13 (41.9%) | 31 |  |  |  |  |
| N3 | 3 (37.5%) | 0 | 5 (62.5%) | 8 |  |  |  |  |
| Sequence of chemotherapy | | | | | 0.242 |  |  |  |
| Post-SOX | 35 (47.3%) | 10 (13.5%) | 29 (39.2%) | 74 |  |  |  |  |
| Peri-SOX | 11 (30.56) | 7 (19.4%) | 18 (50.0%) | 36 |  |  |  |  |
| Gastrectomy |  |  |  |  | 0.491 |  |  |  |
| Total | 29 (46.0%) | 8 (12.7%) | 26 (41.3%) | 63 |  |  |  |  |
| Distal | 17 (36.2%) | 9 (19.2%) | 21 (44.7%) | 47 |  |  |  |  |
| Surgical method |  |  |  |  | 0.231 |  |  |  |
| Open | 16 (39.0%) | 4 (9.8%) | 21 (51.2%) | 41 |  |  |  |  |
| Laparoscopy | 30 (43.5%) | 13 (18.8%) | 26 (37.7%) | 69 |  |  |  |  |
| Surgical complications | | | | | 0.440 |  |  |  |
| No | 42 (44.2%) | 14 (14.7%) | 39 (41.1%) | 95 |  |  |  |  |
| Yes | 4 (26.7%) | 3 (20.0%) | 8 (53.3%) | 15 |  |  |  |  |
| Adverse events of chemotherapy | | | | | 0.377 |  |  |  |
| No | 2 (33.3%) | 0 | 4 (66.67) | 6 |  |  |  |  |
| Yes | 44 (42.3%) | 17 (16.4%) | 43 (41.3%) | 104 |  |  |  |  |

Data presented as n (%).

Abbreviations: BMI: body mass index; ASA: American Society of Anesthesiologists; OR: odds ratio; CI: confidence interval; Ref: reference group.

^A^ Bold *P* values indicating statistical significance (*P* <0.05) according to the Mantel-Haenszel Chi-Squared Tests.

^B^ Bold *P* values indicating statistical significance (*P* <0.05) according to the ordinal logistic regression analysis.

**Table S6**. Univariate and multivariate analyses of associations between clinically relevant factors and changes of physical functioning of LAGC patients before PSM in the 12th month after the initiation of therapy.

| Variables | Changes of physical functioning (%) | | | Total | Univariate analysis  *P* ^A^ | Multivariate analysis | | |
| --- | --- | --- | --- | --- | --- | --- | --- | --- |
|  | Deteriorated | Stable | Improved |  |  | OR | 95% CI | *P* ^B^ |
| Age |  |  |  |  | 0.254 |  |  |  |
| < 60 | 12 (22.6%) | 33 (62.3%) | 8 (15.1%) | 53 |  |  |  |  |
| ≥ 60 | 21 (36.8%) | 30 (52.6%) | 6 (10.4%) | 57 |  |  |  |  |
| Gender |  |  |  |  | 0.576 |  |  |  |
| Male | 23 (30.3%) | 45 (59.2%) | 8 (10.5%) | 76 |  |  |  |  |
| Female | 10 (29.4%) | 18 (52.9%) | 6 (17.7%) | 34 |  |  |  |  |
| BMI |  |  |  |  | 0.384 |  |  |  |
| < 25 | 19 (26.8%) | 41 (57.8%) | 11 (15.4%) | 71 |  |  |  |  |
| ≥ 25 | 14 (35.9%) | 22 (56.4%) | 3 (7.7%) | 39 |  |  |  |  |
| ASA score |  |  |  |  | 0.238 |  |  |  |
| I | 8 (47.1%) | 7 (41.2%) | 2 (11.8%) | 17 |  |  |  |  |
| II | 25 (26.9%) | 56 (60.2%) | 12 (12.9%) | 93 |  |  |  |  |
| Comorbidities |  |  |  |  | 0.787 |  |  |  |
| None | 24 (32.0%) | 42 (56.0%) | 9 (12.0%) | 75 |  |  |  |  |
| ≥ 1 | 9 (25.7%) | 21 (60.0%) | 5 (14.3%) | 35 |  |  |  |  |
| Clinical T stage |  |  |  |  | 0.366 |  |  |  |
| T2 | 3 (18.8%) | 9 (56.2%) | 4 (25.0%) | 16 |  |  |  |  |
| T3 | 16 (32.0%) | 30 (60.0%) | 4 (8.0%) | 50 |  |  |  |  |
| T4 | 14 (31.8%) | 24 (54.6%) | 6 (13.6%) | 44 |  |  |  |  |
| Clinical N stage |  |  |  |  | 0.257 |  |  |  |
| N0 | 13 (30.2%) | 25 (58.1%) | 5 (11.6%) | 43 |  |  |  |  |
| N1 | 15 (48.4%) | 10 (32.3%) | 6 (19.4%) | 31 |  |  |  |  |
| N2 | 2 (25.0%) | 5 (62.5%) | 1 (12.5%) | 8 |  |  |  |  |
| N3 | 13 (30.2%) | 25 (58.1%) | 5 (11.6%) | 43 |  |  |  |  |
| Sequence of chemotherapy | | | | | 0.306 |  |  |  |
| Post-SOX | 24 (32.4%) | 43 (58.1%) | 7 (9.5%) | 74 |  |  |  |  |
| Peri-SOX | 9 (25.0%) | 20 (55.6%) | 7 (19.4%) | 36 |  |  |  |  |
| Gastrectomy |  |  |  |  | 0.086 |  |  |  |
| Total | 24 (38.1%) | 31 (49.2%) | 8 (12.7%) | 63 |  | Ref |  |  |
| Distal | 9 (19.1%) | 32 (68.1%) | 6 (12.8%) | 47 |  | 2.26 | (0.81,6.32) | 0.121 |
| Surgical method |  |  |  |  | 0.898 |  |  |  |
| Open | 12 (29.3%) | 23 (56.1%) | 6 (14.6%) | 41 |  |  |  |  |
| Laparoscopy | 21 (30.4%) | 40 (58.0%) | 8 (11.6%) | 69 |  |  |  |  |
| Surgical complications | | | | | 0.485 |  |  |  |
| No | 29 (30.5%) | 55 (57.9%) | 11 (11.6%) | 95 |  |  |  |  |
| Yes | 4 (26.7%) | 8 (53.3%) | 3 (20.0%) | 15 |  |  |  |  |
| Adverse events of chemotherapy | | | | | **0.044** |  |  |  |
| No | 1 (16.7%) | 2 (33.3%) | 3 (50.0%) | 6 |  | Ref |  |  |
| Yes | 32 (30.8%) | 61 (58.7%) | 11 (10.6%) | 104 |  | 0.08 | (0.01,0.70) | **0.023** |

Data presented as n (%).

Abbreviations: BMI: body mass index; ASA: American Society of Anesthesiologists; OR: odds ratio; CI: confidence interval; Ref: reference group.

^A^ Bold *P* values indicating statistical significance (*P* <0.05) according to the Mantel-Haenszel Chi-Squared Tests.

^B^ Bold *P* values indicating statistical significance (*P* <0.05) according to the ordinal logistic regression analysis.

**Table S7**. Univariate and multivariate analyses of associations between clinically relevant factors and changes of diarrhea of LAGC patients before PSM in the 12th month after the initiation of therapy.

| Variables | Changes of diarrhea (%) | | | Total | Univariate analysis  *P* ^A^ | Multivariate analysis | | |
| --- | --- | --- | --- | --- | --- | --- | --- | --- |
|  | Deteriorated | Stable | Improved |  |  | OR | 95% CI | *P* ^B^ |
| Age |  |  |  |  | 0.205 |  |  |  |
| < 60 | 15 (28.3%) | 29 (54.7%) | 9 (17.0%) | 53 |  |  |  |  |
| ≥ 60 | 22 (38.6%) | 31 (54.4%) | 4 (7.0%) | 57 |  |  |  |  |
| Gender |  |  |  |  | 0.532 |  |  |  |
| Male | 28 (36.9%) | 40 (52.6%) | 8 (10.5%) | 76 |  |  |  |  |
| Female | 9 (26.5%) | 20 (58.8%) | 5 (14.7%) | 34 |  |  |  |  |
| BMI |  |  |  |  | 0.477 |  |  |  |
| < 25 | 21 (29.5%) | 41 (57.8%) | 9 (12.7%) | 71 |  |  |  |  |
| ≥ 25 | 16 (41.0%) | 19 (48.7%) | 4 (10.3%) | 39 |  |  |  |  |
| ASA score |  |  |  |  | 0.670 |  |  |  |
| I | 6 (35.3%) | 8 (47.1%) | 3 (17.6%) | 17 |  |  |  |  |
| II | 31 (33.3%) | 52 (55.9%) | 10 (10.8%) | 93 |  |  |  |  |
| Comorbidities |  |  |  |  | 0.724 |  |  |  |
| None | 24 (32.0%) | 41 (54.7%) | 10 (13.3%) | 75 |  |  |  |  |
| ≥ 1 | 13 (37.1%) | 19 (54.3%) | 3 (8.6%) | 35 |  |  |  |  |
| Clinical T stage |  |  |  |  | 0.685 |  |  |  |
| T2 | 4 (25.0%) | 10 (62.5%) | 2 (12.5%) | 16 |  |  |  |  |
| T3 | 17 (34.0%) | 28 (56.0%) | 5 (10.0%) | 50 |  |  |  |  |
| T4 | 16 (36.4%) | 22 (50.0%) | 6 (13.6%) | 44 |  |  |  |  |
| Clinical N stage |  |  |  |  | 0.291 |  |  |  |
| N0 | 8 (28.6%) | 17 (60.7%) | 3 (10.7%) | 28 |  |  |  |  |
| N1 | 11 (25.6%) | 25 (58.1%) | 7 (16.3%) | 43 |  |  |  |  |
| N2 | 15 (48.4%) | 14 (45.2%) | 2 (6.5%) | 31 |  |  |  |  |
| N3 | 3 (37.5%) | 4 (50.0%) | 1 (12.5%) | 8 |  |  |  |  |
| Sequence of chemotherapy | | | | | 0.893 |  |  |  |
| Post-SOX | 25 (33.8%) | 41 (55.4%) | 8 (10.8%) | 74 |  |  |  |  |
| Peri-SOX | 12 (33.3%) | 19(52.8%) | 5 (13.9%) | 36 |  |  |  |  |
| Gastrectomy |  |  |  |  | **0.021** |  |  |  |
| Total | 28 (44.4%) | 29 (46.0%) | 6 (9.5%) | 63 |  | Ref |  |  |
| Distal | 9 (19.12%) | 31 (66.0%) | 7 (14.9%) | 47 |  | 2.39 | (1.10,5.20) | **0.029** |
| Surgical method |  |  |  |  | 0.429 |  |  |  |
| Open | 16 (39.0%) | 22 (53.7%) | 3 (7.3%) | 41 |  |  |  |  |
| Laparoscopy | 21 (30.4%) | 38 (55.1%) | 10 (14.5%) | 69 |  |  |  |  |
| Surgical complications | | | | | 0.739 |  |  |  |
| No | 31 (32.6%) | 52 (54.7%) | 12 (12.6%) | 95 |  |  |  |  |
| Yes | 6 (40.0%) | 8 (53.3%) | 1 (6.7%) | 15 |  |  |  |  |
| Adverse events of chemotherapy | | | | | **0.030** |  |  |  |
| No | 0 | 4 (66.7%) | 2 (33.3%) | 6 |  | Ref |  |  |
| Yes | 37 (35.6%) | 56 (53.9%) | 11 (10.5%) | 104 |  | 0.248 | (0.05,1.32) | 0.102 |

Data presented as n (%).

Abbreviations: BMI: body mass index; ASA: American Society of Anesthesiologists; OR: odds ratio; CI: confidence interval; Ref: reference group.

^A^ Bold *P* values indicating statistical significance (*P* <0.05) according to the Mantel-Haenszel Chi-Squared Tests.

^B^ Bold *P* values indicating statistical significance (*P* <0.05) according to the ordinal logistic regression analysis.

**Table S8**. Univariate and multivariate analyses of associations between clinically relevant factors and changes of role functioning of LAGC patients before PSM in the 12th month after the initiation of therapy.

| Variables | Changes of role functioning (%) | | | Total | Univariate analysis  *P* ^A^ | Multivariate analysis | | |
| --- | --- | --- | --- | --- | --- | --- | --- | --- |
|  | Deteriorated | Stable | Improved |  |  | OR | 95% CI | *P* ^B^ |
| Age |  |  |  |  | 0.483 |  |  |  |
| < 60 | 17 (32.1%) | 24 (45.3%) | 12 (22.6%) | 53 |  |  |  |  |
| ≥ 60 | 24 (42.1%) | 20 (35.1%) | 13 (22.8%) | 57 |  |  |  |  |
| Gender |  |  |  |  | 0.524 |  |  |  |
| Male | 29 (38.2%) | 32 (42.1%) | 15 (19.7%) | 76 |  |  |  |  |
| Female | 12 (35.3%) | 12 (35.3%) | 10 (29.4%) | 34 |  |  |  |  |
| BMI |  |  |  |  | 0.525 |  |  |  |
| < 25 | 24 (33.8%) | 29 (40.9%) | 18 (25.3%) | 71 |  |  |  |  |
| ≥ 25 | 17 (43.6%) | 15 (38.5%) | 7 (17.9%) | 39 |  |  |  |  |
| ASA score |  |  |  |  | 0.905 |  |  |  |
| I | 7 (41.2%) | 6 (35.3%) | 4 (23.5%) | 17 |  |  |  |  |
| II | 34 (36.6%) | 38 (40.9%) | 21 (22.5%) | 93 |  |  |  |  |
| Comorbidities |  |  |  |  | 0.858 |  |  |  |
| None | 28 (37.3%) | 31 (41.3%) | 16 (21.4%) | 75 |  |  |  |  |
| ≥ 1 | 13 (37.2%) | 13 (37.1%) | 9 (25.7%) | 35 |  |  |  |  |
| Clinical T stage |  |  |  |  | 0.467 |  |  |  |
| T2 | 5 (31.2%) | 7 (43.8%) | 4 (25.0%) | 16 |  |  |  |  |
| T3 | 15 (30.0%) | 23 (46.0%) | 12 (24.0%) | 50 |  |  |  |  |
| T4 | 21 (47.7%) | 14 (31.8%) | 9 (20.5%) | 44 |  |  |  |  |
| Clinical N stage |  |  |  |  | 0.197 |  |  |  |
| N0 | 5 (17.9%) | 17 (60.7%) | 6 (21.4%) | 28 |  | Ref |  |  |
| N1 | 17 (39.5%) | 15 (34.9%) | 11 (25.6%) | 43 |  | 0.40 | (0.08,2.12) | 0.283 |
| N2 | 18 (58.1%) | 6 (19.4%) | 7 (22.5%) | 31 |  | 0.20 | (0.04,1.06) | 0.058 |
| N3 | 1 (12.5%) | 6 (75.0%) | 1 (12.5%) | 8 |  | 0.52 | (0.06,5.00) | 0.574 |
| Sequence of chemotherapy | | | | | 0.355 |  |  |  |
| Post-SOX | 26 (35.1%) | 33 (44.6%) | 15 (20.3%) | 74 |  |  |  |  |
| Peri-SOX | 15 (41.7%) | 11 (30.5%) | 10 (27.8%) | 36 |  |  |  |  |
| Gastrectomy |  |  |  |  | 0.195 |  |  |  |
| Total | 28 (44.5%) | 22 (34.9%) | 13 (20.6%) | 63 |  | Ref |  |  |
| Distal | 13 (27.7%) | 22 (46.8%) | 12 (25.5%) | 47 |  | 1.89 | (0.72,4.94) | 0.195 |
| Surgical method |  |  |  |  | 0.578 |  |  |  |
| Open | 16 (39.0%) | 14 (34.2%) | 11 (26.8%) | 41 |  |  |  |  |
| Laparoscopy | 25 (36.2%) | 30 (43.5%) | 14 (20.3%) | 69 |  |  |  |  |
| Surgical complications | | | | | 0.428 |  |  |  |
| No | 38 (40.0%) | 35 (36.8%) | 22 (23.2%) | 95 |  |  |  |  |
| Yes | 3 (20.0%) | 9 (60.0%) | 3 (20.0%) | 15 |  |  |  |  |
| Adverse events of chemotherapy | | | | | 0.269 |  |  |  |
| No | 1 (16.7%) | 2 (33.3%) | 3 (50.0%) | 6 |  |  |  |  |
| Yes | 40 (38.5%) | 42 (40.3%) | 22 (21.2%) | 104 |  |  |  |  |

Data presented as n (%).

Abbreviations: BMI: body mass index; ASA: American Society of Anesthesiologists; OR: odds ratio; CI: confidence interval; Ref: reference group.

^A^ Bold *P* values indicating statistical significance (*P* <0.05) according to the Mantel-Haenszel Chi-Squared Tests.

^B^ Bold *P* values indicating statistical significance (*P* <0.05) according to the ordinal logistic regression analysis.

**Table S9**. Univariate and multivariate analyses of associations between clinically relevant factors and changes of social functioning of LAGC patients before PSM in the 12th month after the initiation of therapy.

| Variables | Changes of social functioning (%) | | | Total | Univariate analysis  *P* ^A^ | Multivariate analysis | | |
| --- | --- | --- | --- | --- | --- | --- | --- | --- |
|  | Deteriorated | Stable | Improved |  |  | OR | 95% CI | *P* ^B^ |
| Age |  |  |  |  | 0.440 |  |  |  |
| < 60 | 20 (37.7%) | 22 (41.5%) | 11 (20.8%) | 53 |  |  |  |  |
| ≥ 60 | 26 (45.6%) | 17 (29.8%) | 14 (24.6%) | 57 |  |  |  |  |
| Gender |  |  |  |  | 0.418 |  |  |  |
| Male | 30 (39.5%) | 30 (39.5%) | 16 (21.0%) | 76 |  |  |  |  |
| Female | 16 (47.0%) | 9 (26.5%) | 9 (26.5%) | 34 |  |  |  |  |
| BMI |  |  |  |  | 0.395 |  |  |  |
| < 25 | 28 (39.4%) | 24 (33.8%) | 19 (26.8%) | 71 |  |  |  |  |
| ≥ 25 | 18 (46.1%) | 15 (38.5%) | 6 (15.4%) | 39 |  |  |  |  |
| ASA score |  |  |  |  | 0.405 |  |  |  |
| I | 6 (35.3%) | 5 (29.4%) | 6 (35.3%) | 17 |  |  |  |  |
| II | 40 (43.0%) | 34 (36.6%) | 19 (20.4%) | 93 |  |  |  |  |
| Comorbidities |  |  |  |  | 0.753 |  |  |  |
| None | 33 (44.0%) | 25 (33.3%) | 17 (22.7%) | 75 |  |  |  |  |
| ≥ 1 | 13 (37.1%) | 14 (40.0%) | 8 (22.9%) | 35 |  |  |  |  |
| Clinical T stage |  |  |  |  | 0.307 |  |  |  |
| T2 | 7 (43.8%) | 3 (18.7%) | 6 (37.5%) | 16 |  |  |  |  |
| T3 | 19 (38.0%) | 22 (44.0%) | 9 (18.0%) | 50 |  |  |  |  |
| T4 | 20 (45.5%) | 14 (31.8%) | 10 (22.7%) | 44 |  |  |  |  |
| Clinical N stage |  |  |  |  | 0.464 |  |  |  |
| N0 | 14 (50.0%) | 8 (28.6%) | 6 (21.4%) | 28 |  |  |  |  |
| N1 | 13 (30.2%) | 18 (41.9%) | 12 (27.9%) | 43 |  |  |  |  |
| N2 | 14 (45.2%) | 10 (32.2%) | 7 (22.6%) | 31 |  |  |  |  |
| N3 | 5 (62.5%) | 3 (37.5%) | 0 | 8 |  |  |  |  |
| Sequence of chemotherapy | | | | | 0.180 |  |  |  |
| Post-SOX | 33 (44.6%) | 28 (37.8%) | 13 (17.6%) | 74 |  | Ref |  |  |
| Peri-SOX | 13 (36.1%) | 11 (30.6%) | 12 (33.3%) | 36 |  | 2.34 | (0.94,5.81) | 0.067 |
| Gastrectomy |  |  |  |  | **0.024** |  |  |  |
| Total | 23 (36.5%) | 29 (46.0%) | 11 (17.5%) | 63 |  | Ref |  |  |
| Distal | 23 (48.9%) | 10 (21.3%) | 14 (29.8%) | 47 |  | 1.28 | (0.50,3.29) | 0.603 |
| Surgical method |  |  |  |  | 0.979 |  |  |  |
| Open | 17 (41.5%) | 15 (36.5%) | 9 (22.0%) | 41 |  |  |  |  |
| Laparoscopy | 29 (42.0%) | 24 (34.8%) | 16 (23.2%) | 69 |  |  |  |  |
| Surgical complications | | | | | 0.918 |  |  |  |
| No | 40 (42.1%) | 33 (34.7%) | 22 (23.2%) | 95 |  |  |  |  |
| Yes | 6 (40.0%) | 6 (40.0%) | 3 (20.0%) | 15 |  |  |  |  |
| Adverse events of chemotherapy | | | | | 0.540 |  |  |  |
| No | 3 (50.0%) | 0 | 3 (50.0%) | 6 |  |  |  |  |
| Yes | 43 (41.4%) | 39 (37.5%) | 22 (21.1%) | 104 |  |  |  |  |

Data presented as n (%).

Abbreviations: BMI: body mass index; ASA: American Society of Anesthesiologists; OR: odds ratio; CI: confidence interval; Ref: reference group.

^A^ Bold *P* values indicating statistical significance (*P* <0.05) according to the Mantel-Haenszel Chi-Squared Tests.

^B^ Bold *P* values indicating statistical significance (*P* <0.05) according to the ordinal logistic regression analysis.

**Table S10**. Univariate and multivariate analyses of associations between clinically relevant factors and changes of reflux symptom of LAGC patients before PSM in the 12th month after the initiation of therapy.

| Variables | Changes of reflux symptom (%) | | | Total | Univariate analysis  *P* ^A^ | Multivariate analysis | | |
| --- | --- | --- | --- | --- | --- | --- | --- | --- |
|  | Deteriorated | Stable | Improved |  |  | OR | 95% CI | *P* ^B^ |
| Age |  |  |  |  | 0.438 |  |  |  |
| < 60 | 15 (28.3%) | 15 (28.3%) | 23(43.4%) | 53 |  |  |  |  |
| ≥ 60 | 20 (35.1%) | 19 (33.3%) | 18(31.6%) | 57 |  |  |  |  |
| Gender |  |  |  |  | 0.609 |  |  |  |
| Male | 22 (28.9%) | 24 (31.6%) | 30 (39.5%) | 76 |  |  |  |  |
| Female | 13 (38.2%) | 10 (29.4%) | 11 (32.4%) | 34 |  |  |  |  |
| BMI |  |  |  |  | 0.152 |  |  |  |
| < 25 | 27 (38.0%) | 19 (26.8%) | 25 (35.2%) | 71 |  | Ref |  |  |
| ≥ 25 | 8 (20.5%) | 15 (38.5%) | 16 (41.0%) | 39 |  | 1.61 | (0.78,3.35) | 0.200 |
| ASA score |  |  |  |  | 0.190 |  |  |  |
| I | 7 (41.2%) | 7 (41.2%) | 3 (17.6%) | 17 |  | Ref |  |  |
| II | 28 (30.1%) | 27 (29.0%) | 38 (40.9%) | 93 |  | 2.01 | (0.758,5.35) | 0.160 |
| Comorbidities |  |  |  |  | 0.150 |  |  |  |
| None | 27 (36.0%) | 19 (25.3%) | 29 (38.7%) | 75 |  | Ref |  |  |
| ≥ 1 | 8 (22.8%) | 15 (42.9%) | 12 (34.3%) | 35 |  | 0.90 | (0.50,2.23) | 0.897 |
| Clinical T stage |  |  |  |  | 0.978 |  |  |  |
| T2 | 5 (31.3%) | 6 (37.5%) | 5 (31.2%) | 16 |  |  |  |  |
| T3 | 16 (32.0%) | 15 (30.0%) | 19 (38.0%) | 50 |  |  |  |  |
| T4 | 14 (31.8%) | 13 (29.6%) | 17 (38.6%) | 44 |  |  |  |  |
| Clinical N stage |  |  |  |  | 0.697 |  |  |  |
| N0 | 7 (25.0%) | 10 (35.7%) | 11 (39.3%) | 28 |  |  |  |  |
| N1 | 14 (32.6%) | 12 (27.9%) | 17 (39.5%) | 43 |  |  |  |  |
| N2 | 13 (41.9%) | 9 (29.0%) | 9 (29.0%) | 31 |  |  |  |  |
| N3 | 1 (12.5%) | 3 (37.5%) | 4 (50.0%) | 8 |  |  |  |  |
| Sequence of chemotherapy | | | | | 0.355 |  |  |  |
| Post-SOX | 22 (29.7%) | 21 (28.4%) | 31 (41.9%) | 74 |  |  |  |  |
| Peri-SOX | 13 (36.1%) | 13 (36.1%) | 10 (27.8%) | 36 |  |  |  |  |
| Gastrectomy |  |  |  |  | 0.694 |  |  |  |
| Total | 22 (34.9%) | 18 (28.6%) | 23 (36.5%) | 63 |  |  |  |  |
| Distal | 13 (27.7%) | 16 (34.0%) | 18 (38.3%) | 47 |  |  |  |  |
| Surgical method |  |  |  |  | 0.599 |  |  |  |
| Open | 15 (36.6%) | 13 (31.7%) | 13(31.7%) | 41 |  |  |  |  |
| Laparoscopy | 20 (29.0%) | 21 (30.4%) | 28 (40.6%) | 69 |  |  |  |  |
| Surgical complications | | | | | 0.467 |  |  |  |
| No | 33 (34.7%) | 26 (27.4%) | 36 (37.9%) | 95 |  |  |  |  |
| Yes | 2 (13.3%) | 8 (53.4%) | 5 (33.3%) | 15 |  |  |  |  |
| Adverse events of chemotherapy | | | | | 0.869 |  |  |  |
| No | 1 (16.7%) | 4 (66.6%) | 1 (16.7%) | 6 |  |  |  |  |
| Yes | 34 (32.7%) | 30 (28.8%) | 40 (38.5%) | 104 |  |  |  |  |

Data presented as n (%).

Abbreviations: BMI: body mass index; ASA: American Society of Anesthesiologists; OR: odds ratio; CI: confidence interval; Ref: reference group.

^A^ Bold *P* values indicating statistical significance (*P* <0.05) according to the Mantel-Haenszel Chi-Squared Tests.

^B^ Bold *P* values indicating statistical significance (*P* <0.05) according to the ordinal logistic regression analysis.
